# A low-cost cellulose-based composite as an efficient solid phase extraction sorbent for the determination of antibiotics in water

**DOI:** 10.1039/d5ra02296g

**Published:** 2025-09-10

**Authors:** Damilare Olorunnisola, Chidinma G. Olorunnisola, Christina Günter, Chukwunonso P. Okoli, Martins O. Omorogie, Emmanuel I. Unuabonah, Harshadrai M. Rawel, Andreas Taubert

**Affiliations:** a Institute of Chemistry, University of Potsdam D-14476 Potsdam Germany ataubert@uni-potsdam.de; b Institute of Nutritional Science, University of Potsdam 14558 Nuthetal Potsdam Germany rawel@uni-potsdam.de; c African Centre of Excellence for Water and Environment Research (ACEWATER), Redeemer's University PMB 230 Ede Osun State Nigeria; d Department of Chemical Sciences, Redeemer's University PMB 230 Ede Osun State Nigeria; e Institute of Geosciences, University of Potsdam D-14476 Potsdam Germany; f Department of Chemistry, Alex Ekwueme Federal University Ndufu-Alike Ebonyi State Nigeria

## Abstract

Pharmaceutical pollutants in water pose significant environmental and public health risks, particularly in regions with limited monitoring capabilities. This study presents a low-cost, cellulose-based solid-phase extraction (SPE) adsorbent (C/PVPP/MDI) for the determination of five antibiotics: tetracycline (TET), ampicillin (AMP), sulfamethoxazole (SMX), penicillin V (PEN V), and chloramphenicol (CAP) in water. The adsorbent was synthesized by cross-linking cellulose with poly(vinyl-polypyrrolidone) (PVPP) and 4,4-methylenebisphenyldiisocyanate (MDI). It exhibited excellent analytical performance with low detection limits (0.03–2.07 ng L^−1^), strong linearity (*R*^2^ > 0.99), and high recoveries (84.8–97.6%) in both tap and river water, comparable to a commercial hydrophilic–lipophilic balance (HLB) adsorbent (87.0–97.3%). Additionally, the C/PVPP/MDI adsorbent was reusable for up to five cycles without significant performance loss and costs approximately 50% less than commercial alternatives. These findings demonstrate the potential of C/PVPP/MDI as a sustainable and affordable SPE material for environmental monitoring of antibiotic contaminants, particularly in developing countries where access to commercial materials is limited.

## Abstract 2

A low-cost, cellulose-based solid-phase extraction adsorbent (C/PVPP/MDI) for the determination of five antibiotics (tetracycline, ampicillin, sulfamethoxazole, penicillin V, and chloramphenicol) in water is presented.

## Graphical abstract

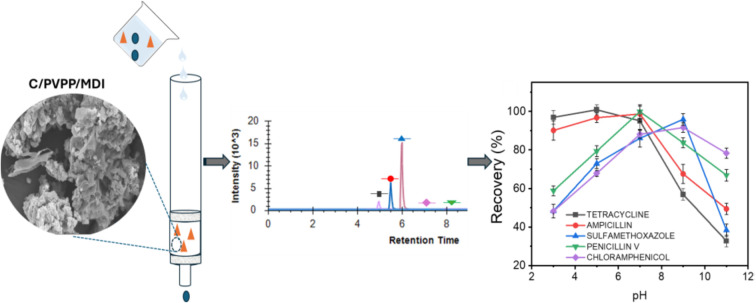

## 1. Introduction

Pharmaceuticals are emerging environmental pollutants that have significantly affected global freshwater availability and their presence in water bodies is becoming an increasing ecological concern. With over 4000 compounds used therapeutically to treat humans and animals,^1^ their continuous use has led to persistent contamination of aquatic environments through sewage discharge, agricultural runoff, industrial effluents, and improper disposal.^2,3^ These contaminants have been linked to various ecological and health risks, including endocrine disruption, antibiotic resistance, and organ toxicity in both aquatic organisms and humans.^4–7^

**Fig. 1:**
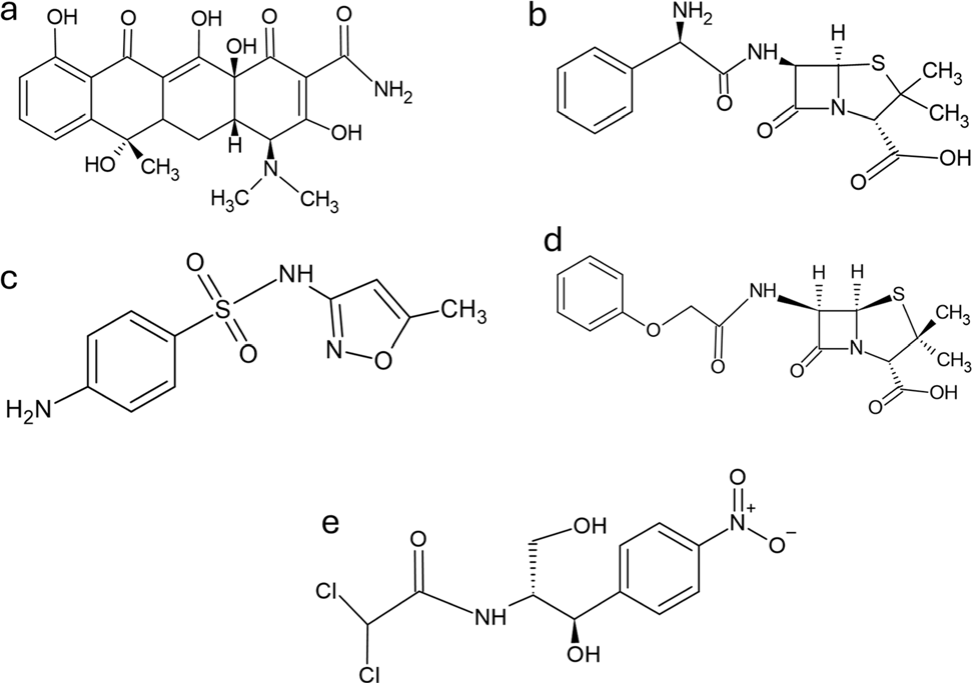
Chemical structure of (a) tetracycline (TET) (b) ampicillin (AMP) (c) sulfamethoxazole (SMX) (d) penicillin V (PEN V) and (e) chloramphenicol (CAP).

Among pharmaceuticals, antibiotics are of particular concern due to their role in the growing crisis of antimicrobial resistance, posing a serious threat to public health.^8,9^ Among the antibiotics, tetracycline (TET, Fig. 1a), ampicillin (AMP, Fig. 1b), sulfamethoxazole (SMX, Fig. 1c), penicillin V (PEN V, Fig. 1d) and chloramphenicol (CAP, Fig. 1e) are common drugs found in environmental waters globally, especially in Africa.^3,10,11^ These drugs are widely used in human and veterinary medicine, with applications across agriculture, livestock, and healthcare.^10,12–15^ Their persistence in aquatic environments is driven by incomplete metabolism and widespread discharge *via* urine, faeces, and industrial waste, leading to significant environmental accumulation and potential ecological harm.

Currently, there is still a significant knowledge gap regarding the occurrence and distribution of these antibiotics in aquatic environments across Africa.^16^ Alarmingly, recent projections indicate that Sub-Saharan Africa is likely to emerge as a major global hotspot for surface water pollution in the near future.^17^ Consequently, one of the most pressing challenges for developing countries is the determination and monitoring of these antibiotics, especially at low concentrations in environmental waters.^2^

Solid-phase extraction (SPE) is an efficient sample preparation technique widely used for water quality analysis due to its high enrichment efficiency, low solvent consumption, and compatibility with analytical instruments.^18^ However, its effectiveness depends largely on the choice of adsorbent. While advanced materials such as hydrophilic–lipophilic balance (HLB) sorbents,^19^ molecularly imprinted polymers,^20^ metal–organic frameworks^21^ and magnetic nanoparticles^22^ have been explored, their high cost, limited selectivity, and poor reusability hinder broad application, particularly in resource-limited settings. Consequently, commercial HLB adsorbents such as Oasis HLB are widely used in developed countries for antibiotic monitoring in water due to their high extraction efficiency; often achieving recoveries between 75–98% for a broad spectrum of pharmaceutical contaminants.^23,24^ However, their cost remains a significant barrier, with a 100-cartridge pack costing over €430 excluding shipping (https://www.analytics-shop.com/de/wtwat094226 Accessed 21st July, 2025), limiting their widespread use in developing countries, particularly in Africa, where water monitoring is often lacking.^2^ This financial constraint highlights the urgent need for affordable and effective SPE adsorbents capable of selectively pre-concentrating antibiotics in aquatic systems.

Despite their challenges, biopolymers like cellulose have been extensively studied as adsorbents for a wide range of pollutants due to their abundance, low cost, renewable nature, and tunable surface chemistry. Numerous studies have employed cellulose and its derivatives for the adsorption of dyes, heavy metals, and pharmaceutical residues from aqueous media, often highlighting its good chemical stability and selectivity for aromatic compounds.^18,25–28^ However, the majority of these studies focus on batch adsorption processes rather than analytical sample preparation. In contrast, the use of cellulose-based materials as solid-phase extraction (SPE) adsorbents for trace-level analysis of pharmaceuticals in water remains limited and underexplored. In our previous study, we reported the development and performance of cross-linked cellulose with 4,4-methylenebisphenyldiisocyanate (MDI) as SPE adsorbent for the determination of hydrophobic pollutants, specifically for chloramphenicol (CAP) and bisphenol A (BPA) in water.^15^ The adsorbent shows good analytical efficiency with a low limit of detection (LOD), 71.9 ng L^−1^ for CAP and 10 ng L^−1^ for BPA, and achieves recovery rates comparable to those obtained using commercial HLB cartridges.

Building on this foundation, the objective of the present study is to develop a low-cost, broad spectrum and recyclable SPE sorbent by modifying cellulose with poly(vinyl-polypyrrolidone) (PVPP) and MDI, and to evaluate the performance of the resulting composite (C/PVPP/MDI) for detecting five commonly used hydrophilic and hydrophobic antibiotics (TET, AMP, SMX, PEN V, and CAP) in water. The novelty of this work lies in the dual functionalization strategy: PVPP, a crosslinked polymer with affinity for both polar and nonpolar compounds, enhances hydrophilicity and facilitates interactions *via* hydrogen bonding and electrostatic forces, while MDI, a reactive hydrophobic cross-linker, improves the structural rigidity and reusability of the composite. This combination yields a broad-spectrum sorbent with stable performance, enabling trace-level recovery of pharmaceutical pollutants, especially in resource-limited settings, at a fraction of the cost of commercial HLB sorbents. The five model antibiotics were selected based on their global use and frequent detection in environmental waters, particularly in regions where affordable water monitoring solutions are urgently needed.^13,14,29^

## 2. Experimental section

### 2.1. Materials and reagents

4,4–Methylenebisphenyldiisocyanate (98%), cellulose microcrystalline powder (98%), polyvinylpolypyrrolidone (98%), ampicillin (98%), tetracycline (98%), sulfamethoxazole (98%), chloramphenicol (98%), penicillin V (98%), *N*,*N*-dimethylformamide (≥99%), methanol (≥99%), acetonitrile (≥99%), ethyl acetate (≥99%, all Sigma-Aldrich), and Oasis HLB SPE cartridges (Waters, 200 mg) were used as purchased. These reagents were used without further purification. Primary stock solutions of the analytes (1 mg mL^−1^) were prepared using a 1 : 1 mixture of ethanol and water, and kept refrigerated at 4 °C. Working solutions were freshly prepared by diluting the stock solutions with ultrapure water immediately before analysis.

### 2.2. Synthesis of SPE adsorbents

Different SPE adsorbents were synthesized by modifying a previously reported preparation method.^15^ The composite adsorbent was prepared by dissolving 2.0 g of cellulose in 20 mL of dry dimethylformamide (DMF, boiling point 153 °C) at 70 °C. Then, 2.0 g of poly(vinyl-polypyrrolidone) (PVPP) was added, and the mixture was stirred for 15 min at 70 °C. Subsequently, 2.0 g of 4,4-methylenebisphenyldiisocyanate (MDI) was introduced into the suspension, which was then stirred continuously at 70 °C for 6 hours. The resulting slurry was aged overnight in an oven at 70 °C. The resulting precipitate was then washed with ultra-pure water until neutral pH, dried to constant weight at 40 °C, ground and stored for future use. This composite is denoted C/PVPP/MDI throughout the manuscript.

Other adsorbents were synthesized by separately reacting equal masses of cellulose + MDI and only cellulose + PVPP under the same conditions as stated above, 70 °C in 20 mL of dry DMF with continuous stirring for 6 h. Purification and workup were done as described in the above procedure. The adsorbent prepared with cellulose + MDI is denoted C/MDI, and that prepared with cellulose + PVPP is denoted C/PVPP. A neat PVPP/MDI adsorbent was also synthesized according to the procedure described above to produce control materials.

### 2.3. Characterization

The point of zero charge, pH_pzc_, was determined *via* the salt addition method providing the net surface charge of the adsorbents.^12^ Powder X-ray diffraction (PXRD) was done on a PANalytical EmpyreanPowder X-ray diffractometer (Malvern, UK) in a Bragg–Brentano geometry equipped with a PIXcel1D detector using Cu K_α_ radiation (*λ* = 1.5419 Å) operating at 40 kV and 40 mA; *θ*/*θ* scans were run from 4–70° 2*θ* with a step size of 0.0131°, and a sample rotation time of 1 s. Scanning electron microscopy was done on a JEOL JSM-6510 SEM (Freising, Germany) equipped with an Oxford Instruments INCAx-act detector (energy dispersive X-ray detector (EDX), Oxford Instruments, High Wycombe, UK). Elemental analysis was done on an Elementar Vario EL III (Langenselbold, Germany) in duplicate. Attenuated total reflectance Fourier transform infrared (ATR-FTIR) spectroscopy was done on a Nicolet iS5 (Thermo Scientific, Waltham, MA, USA, iD7 ATR unit with a diamond crystal, resolution of 4 cm^−1^, 32 scans, from 400 to 4000 cm^−1^). Nitrogen sorption measurements were recorded on a Microtrac Belsorp MAX (formerly from BEL Instruments) and Microtrac Belsorp MINI X. The samples were prepared/activated in the Microtrac BELPREP VAC III vacuum degasser (24 hours under vacuum at 150 °C). The specific surface area was calculated using the Brunauer–Emmett–Teller (BET) method. The average pore sizes were estimated from the adsorption branch of the isotherm using the Barrett–Joyner–Halenda (BJH) method. The pore volume was determined at *P*/*P*_0_ > 0.99. Thermogravimetric analysis (TGA) was done on a Mettler Toledo (TGA/DSC 3+) 700 °C under nitrogen (50 mL min^−1^ gas flow) with a heating rate of 10 K min^−1^. LC-MS/MS analysis was performed using an Agilent G6470A Series Triple Quad LC/MS (Agilent Technologies Sales & Services GmbH & Co. KG, Waldbronn, Germany) and HPLC (Agilent Infinity 1260 System, binary pump, multicolumn thermostat, vial sampler VL, UV-Vis Dual Wavelength Detector set at 470, 538 nm).

### 2.4. Adsorbent screening

A comparative study was carried out with treated cellulose as a control to evaluate the performance of the SPE adsorbents for TET, AMP, SMX, PEN V, and CAP adsorption. Simple time-profile experiments were carried out using 300 mg of each SPE adsorbent packed into an empty 6 mL polypropylene cartridge containing polyethylene frits at the top and bottom of the cartridge to hold the adsorbent in place. The cartridges were subsequently connected to an SPE manifold with a vacuum pump. Next, the cartridges were conditioned with 5 mL methanol to leach impurities, followed by 5 mL of ultra-pure water to equilibrate the phase. Then, 150 mL of sample volume containing 5 ng mL^−1^ of each analyte was passed through the pre-conditioned cartridges at 5 mL min^−1^. Samples were collected at different time intervals and analyzed with LC-MS/MS.

### 2.5. Extraction, optimization and regeneration procedure

Due to the better performance of C/PVPP/MDI over the other materials described in the materials section above, these studies were only done with C/PVPP/MDI. The SPE cartridges were assembled by packing C/PVPP/MDI powder in a 6 mL polypropylene cartridge. Next, a 100 mL sample volume containing 0.025 ng mL^−1^ of the analytes of interest was passed through the pre-conditioned C/PVPP/MDI cartridge at an appropriate flow rate (Section 3.2). Then, the cartridge was washed with 5 mL of ultrapure water to remove physisorbed substances and dried for 10 min. The analytes adsorbed on the adsorbent were then eluted with the suitable elution solvent (Section 3.2). Thereafter, the eluate was evaporated to dryness in a vacuum oven and reconstituted in 0.5 mL of 1 : 1 ethanol and water for LC-MS/MS analysis. All the experiments (SPE extraction and LC-MS/MS analysis) were carried out in triplicates.

The effects of several operating variables that can affect extraction recovery were studied. The effect of adsorbent dose (100–400 mg), pH (3.0, 5.0, 7.0, 9.0 and 11.0 adjusted with 0.1 M HCl and 0.1 M NaOH), flow rate (1.5, 3.0 and 5.0 mL min^−1^), sample volume (100, 250 and 500 mL), elution solvents (methanol, acetonitrile, ethyl acetate, isopropanol, dichloromethane, mixture and acidified mixture of solvents), elution solvent volume (2.0, 5.0 and 8.0 mL), and elution flow rate (1.5, 3.0 and 5.0 mL min^−1^) on the extraction of 0.025 ng mL^−1^ of TET, AMP, SMX, PEN V and CAP were investigated. Extraction recovery (%*R*, eqn (S1), SI), calculated from the calibration data of the five antibiotics (Fig. S1a–e, SI) was used to evaluate the effect of the parameters on the SPE performance. To assess the reusability of the C/PVPP/MDI adsorbent, the used SPE material was regenerated with 10 mL of methanol, followed by 10 mL of ultrapure water. The CMDI-1 cartridge was then air-dried for 30 minutes before being reused in the subsequent extraction cycle.

### 2.6. Chromatography and mass spectrometry

The separation of the antibiotics was achieved using a Poroshell 120 EC-C18 (3.0 × 50 mm, 2.7 μm, Agilent technologies) column. The sample injection volume was 10 μL at a flow rate of 0.6 mL min^−1^. The gradient mobile phase consisted of water with 0.1% formic acid (solution A) and methanol with 0.1% formic acid (solution B). The elution gradient for the mobile phase is as follows: 0–0.5 min; 90% solution A, 0.5–4 min; 90–70% solution A, 4–6 min; 70–30% solution A; 6–8 min; 30% solution A; 8–9 min; 10% solution A. The difference to 100% is solution B.

Mass spectrometric analysis was performed using a triple quadrupole instrument operated in selected reaction monitoring (SRM) mode. Multiple reaction monitoring (MRM) was employed under both positive and negative electrospray ionization (ESI) conditions, depending on the analyte, to ensure optimal detection sensitivity. Table S1 outlines the instrumental parameter settings used for mass spectrometry conditions. These results are presented in Table S2.

### 2.7. Method validation and analytical performance

Quality assurance parameters such as limit of detection (LOD), limit of quantification (LOQ), linear range, recovery and precision were used to assess the performance of the developed pre-concentration method (see details in S2.0, SI). Calibration curves were constructed by plotting peak area against the corresponding analyte concentration, and linearity was evaluated using the correlation coefficient (*R*^2^) of the resulting linear regression (Fig. S2a–e, SI). These validation parameters were assessed per standard guidelines for laboratory validation of analytical methods targeting trace-level organic contaminants.^18,30^ Precision of the method was evaluated in terms of intra- and inter-day assay variability using 100 mL of aqueous samples spiked with the analytes at three different concentration levels. Each analysis was done in triplicate on the same day and on three consecutive days.

### 2.8. Environmental sample analysis

Environmental water samples including tap water (groundwater) and river water (surface water)††according to the Gesetz zur Ordnung des Wasserhaushalts (Wasserhaushaltsgesetz – WHG), § 25 Gemeingebrauch. were used to assess the feasibility of our approach for the extraction of the five analytes of interest. The result was also compared with data obtained from the commercial HLB cartridge. The river water samples were collected from the Nuthe river at the Nuthebrücke (Humboldtring), Potsdam, Germany, while the tap water samples were collected at the University of Potsdam, Germany. Water samples were collected in 500 mL amber glass bottles that had been pre-cleaned with methanol and ultrapure water, and subsequently rinsed on-site with the respective water sample. After collection, the samples were transported to the laboratory and stored at 4 °C until SPE analysis was conducted. Each 100 mL water sample was spiked with a known concentration of the target analytes and loaded onto the C/PVPP/MDI SPE cartridge at a flow rate of 5 mL min^−1^. For control purposes, unspiked water samples were also processed under identical conditions using the same SPE protocol.

## 3. Results and discussion

### 3.1. Characterization

**Fig. 2:**
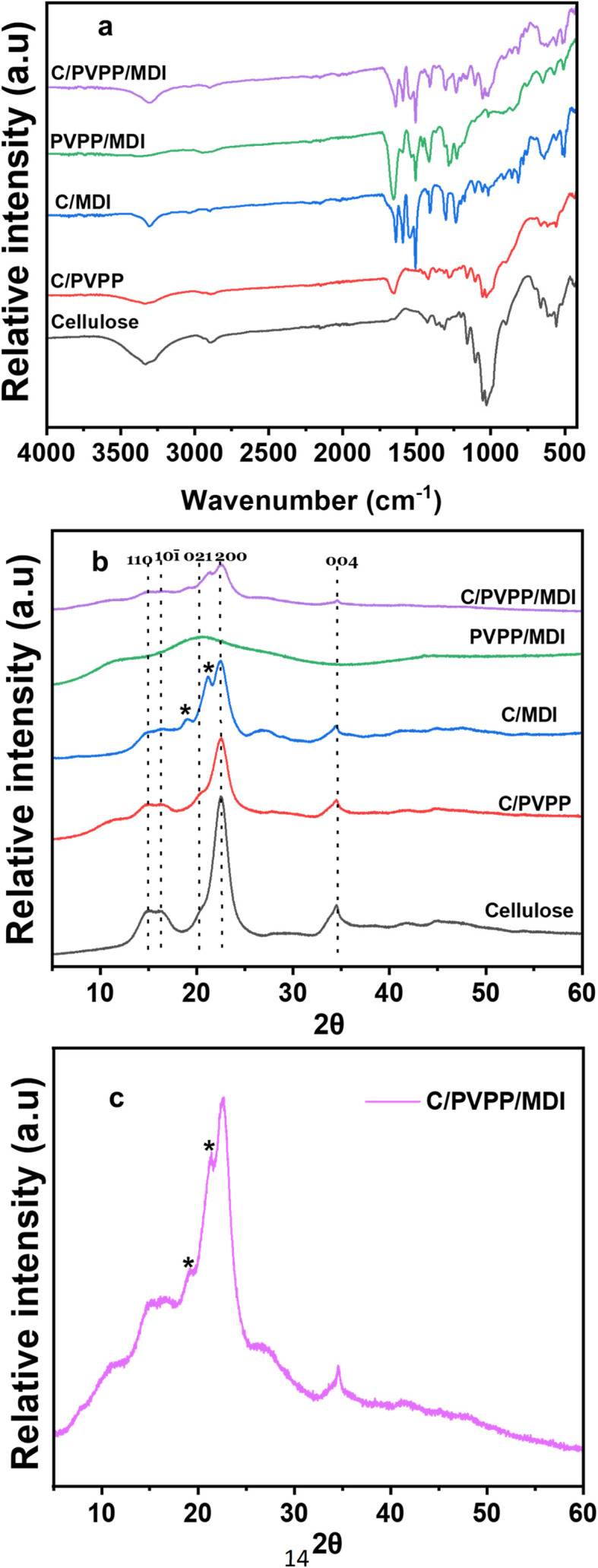
(a) ATR-FTIR spectra and (b) XRD patterns of cellulose and the adsorbents (c) XRD pattern of C/PVPP/MDI. * indicates new reflections from MDI cross-linking. See text for details.

The ATR-FTIR spectra of cellulose and the different modified composites are shown in Fig. 2a. The bands at 3200–3600 and 2842–2908 cm^−1^ are assigned to O–H and C–H stretching vibrations of cellulose, respectively. They are found in the spectra of all materials except for PVPP/MDI. Additionally, the absorption bands observed at 1060, 1030, and 1165 cm^−1^ are attributed to C–OH stretching vibrations associated with the secondary and primary alcohol groups in cellulose, as well as the asymmetric ring breathing vibration characteristic of cellulose.^15^ Compared with cellulose, the bands at 1291, 1426, 1666 and 2953 cm^−1^ in C/PVPP, PVPP/MDI and C/PVPP/MDI are assigned to C–N, C=C, C=O and C–H stretching vibrations of PVPP, respectively.^31^ For both C/MDI and C/PVPP/MDI, the absorption band at 3304 cm^−1^ is attributed to the N–H stretching vibration of aromatic urethane groups. The band at 1510 cm^−1^ corresponds to N–H bending in secondary amides (amide II),^32^ while the signal at 1649 cm^−1^ arises from C=O stretching vibrations in secondary amides (amide I). Additionally, the bands at 1595, 3029, and 1406 cm^−1^ are associated with the benzene ring vibrations in MDI, aromatic C–H stretching, and C–C skeletal vibrations, respectively.^15^ Furthermore, the O–H band in cellulose (3300–3500 cm^−1^) is shifted in C/MDI (3220–3385 cm^−1^) and C/PVPP/MDI (3205–3413 cm^−1^). These shifts in absorption bands suggest molecular interactions among MDI, PVPP, and cellulose, likely involving the disruption or rearrangement of hydrogen bonding between cellulose's glycosidic linkages and hydroxyl groups, as a result of incorporating reactive functional groups from MDI and PVPP. This interaction may involve the formation of new hydrogen bonds or other types of secondary bonding, altering the molecular dynamics and possibly affecting the material's mechanical or thermal properties.^33^ Overall, the new functional groups introduced to the hybrid material from MDI and PVPP suggest successful modification and cross-linking.

Fig. 2b shows representative XRD patterns obtained from the adsorbents, while Fig. 2c shows a clearer XRD pattern of C/PVPP/MDI. The reflections at 2*θ* = 15.1°, 16.3°, 20.3°, 22.6° and 34.6° which are characteristic reflections of cellulose corresponding to the lattice planes 110, 101̄, 021, 200 and 004,^34,35^ are visible in all the adsorbents except PVPP/MDI. However, there seems to be a reduction of intensity in the cellulose reflection at 22.6° across the adsorbents. Furthermore, C/PVPP, PVPP/MDI and C/PVPP/MDI show a broad reflection at around 11° due to the amorphous nature of PVPP.^36^ The XRD pattern of C/MDI and C/PVPP/MDI adsorbents show new features which could be attributed to MDI crosslinking at 2*θ* = 19.1° and 26.9°. In general, considering the XRD patterns for pristine cellulose and cross-linked adsorbents, it is observed that the crosslinking process had minimal impact on the crystalline structure of cellulose, as previously reported by several authors.^37,38^ However, we do observe two additional reflections at 19.1° and 26.9°, but can't currently assign these reflections to a specific structure in the cross-linked material. Results from both ATR-FTIR and XRD analysis confirm the successful modification of cellulose with MDI and PVPP, which stems from the introduction of new functional groups into the hybrid materials.

**Fig. 3:**
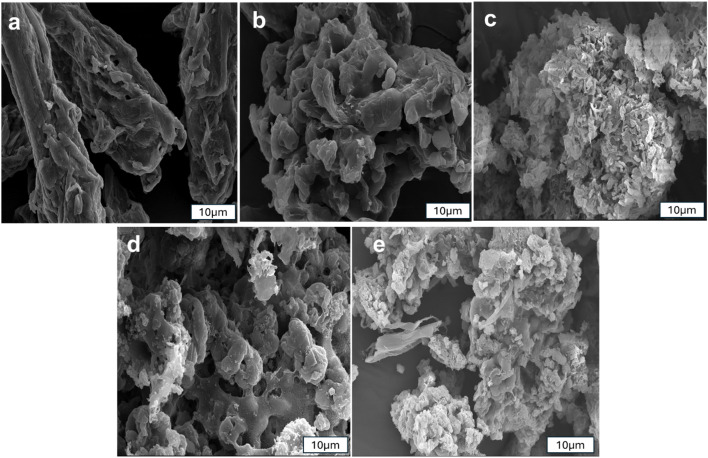
SEM images of (a) cellulose (b) C/PVPP (c) PVPP/MDI (d) C/MDI and (e) C/PVPP/MDI.

Fig. 3 shows scanning electron microscopy (SEM) images of the adsorbents. The morphology of cellulose (Fig. 3a) shows particles with rod-like shape which are agglomerated to form a large structure. The introduction of PVPP (Fig. 3b, c and e) appears to create some pores in the material. Additionally, C/MDI and C/PVPP/MDI (Fig. 3d–e) show dispersed flake-like particles (presumably MDI) deposited on the cellulose/PVPP structure. This observation is similar to previous data.^15^

Table 1 shows the elemental composition of the adsorbents determined *via* elemental analysis (EA). The addition of PVPP and MDI to the cellulose starting material generally leads to an overall increase in the carbon content in C/PVPP, C/MDI and C/PVPP/MDI, as expected from the calculated carbon content in these materials. Also, EA shows that C/PVPP, C/MDI and C/PVPP/MDI contain a significant fraction of nitrogen after the addition of MDI or PVPP which is absent in pristine cellulose. PVPP/MDI contains the highest amount of nitrogen. Hence, more nitrogen is observed in C/PVPP/MDI composite in comparison to C/PVPP and C/MDI due to the addition of two nitrogen-containing components (MDI and PVPP) to the composite. The C/H atomic ratio is an indicator of aromaticity with higher carbon content indicating higher number of aromatic groups The order of C/H atomic ratio for the adsorbents is C/MDI > PVPP/MDI > C/PVPP/MDI > cellulose > C/PVPP. These observations further support the successful introduction of aromatic groups into the chemical structure of cellulose as earlier confirmed with IR spectroscopy.

**Table 1:** Data from EA and nitrogen sorption

Sample	Elements (% composition)					*S*_BET_ (m^2^ g^−1^)	Pore volume (cm^3^ g^−1^)	Pore diameter (nm)
	C	H	O	N	C/H			
Cellulose	42.12	6.29	51.59	—	6.69	1.69	0.39	42.27
C/PVPP	50.77	7.94	35.47	5.82	6.39	0.56	0.13	44.12
PVPP/MDI	62.16	7.70	18.60	11.54	8.05	4.30	0.99	31.57
C/MDI	56.04	6.48	31.93	5.55	8.65	26.06	0.10	15.83
C/PVPP/MDI	56.74	7.60	35.66	7.52	7.47	20.56	0.08	15.12

Table 1 also summarizes the data obtained from the nitrogen sorption experiments. The surface areas of cellulose, C/PVPP and PVPP/MDI are below 10 m^2^ g^−1^, which are insignificant. However, C/MDI and C/PVPP/MDI show improved surface area. In comparison to C/MDI, a slight decrease in the surface area of the C/PVPP/MDI is observed. This could be due to the introduction of PVPP in the composite. In addition, C/MDI and C/PVPP/MDI show a distinctive type II isotherm (Fig. S3, SI) representing unrestricted monolayer-multilayer adsorption.

**Fig. 4:**
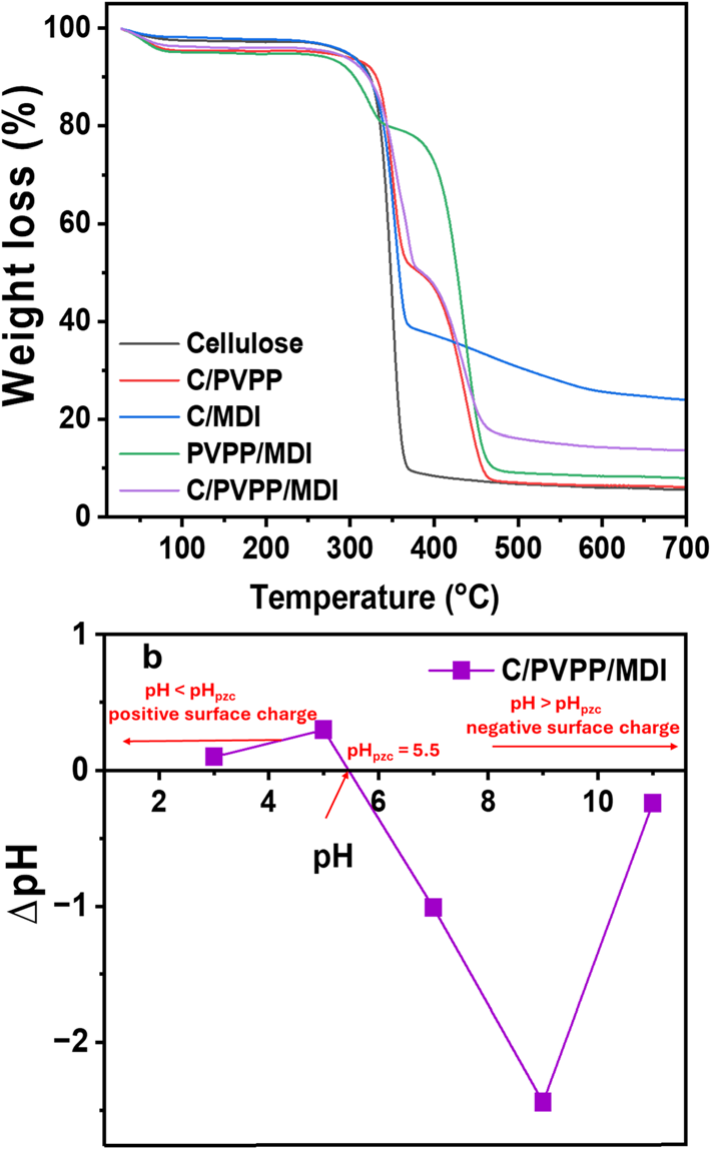
(a) TGA data of all adsorbents and (b) pH_pzc_ plot obtained for C/PVPP/MDI.

Fig. 4a shows representative thermogravimetric analysis (TGA) profiles for all synthesized materials. An initial weight loss occurring between 25 °C and 105 °C is observed across all adsorbents, which is attributed to the evaporation of physically adsorbed moisture. The second weight loss is observed between 255 to 375 °C with a weight loss of *ca.* 43, 45, 58 and 86% for C/PVPP, C/PVPP/MDI, C/MDI and cellulose, respectively. This is attributed to thermal decomposition and depolymerization of cellulose.^38^ The third weight loss corresponds to the thermal degradation of cellulose, characterized by rapid depolymerization of carbonaceous residues occurring above 370 °C, with complete pyrolysis typically reached by around 700 °C. However, the third decomposition stage occurs between *ca.* 368 to 476 °C for C/PVPP and C/PVPP/MDI and between *ca.* 366 to 590 °C for C/MDI. This is due to the decomposition of PVPP, MDI and cellulose carbonaceous matter.^31,39^ The final weight loss between 476 to 700 °C for C/PVPP and C/PVPP/MDI and 590 to 700 °C for C/MDI corresponds to the complete pyrolysis of the adsorbents. Generally, C/PVPP and C/PVPP/MDI show very similar TGA data, but the TGA data of C/PVPP/MDI show a higher residual mass at higher temperatures.

Fig. 4b shows the point of zero charge, pH_pzc_, of C/PVPP/MDI. This allows for the determination of the net surface charge density of the adsorbents at different pH values. The information provided by the pH_pzc_ can be used to adjust the adsorbent surface charge for optimized extraction and adsorption of pollutants.^15^ The pH_pzc_ of C/PVPP/MDI is 5.5. This indicates that the net surface charge of C/PVPP/MDI becomes increasingly positive when the solution pH falls below its point of zero charge (pH_p_zc), and shifts to a negative charge as the pH rises above this value.

C/PVPP/MDI adsorbent has shown the best extraction efficiency more than the other adsorbents in the preliminary test (Fig. S4, SI), hence, it is now the focus adsorbent. Also, C/PVPP/MDI has shown the highest removal rate among all adsorbents studied for the five analytes of interest under consideration.

### 3.2. Effect of operational parameters on C/PVPP/MDI-SPE conditions

C/PVPP/MDI has been employed successfully to extract TET, AMP, SMX, CAP and PEN V from synthetic and real polluted water samples. Analytical recovery (R%) (eqn (S1), SI) is used to evaluate all experimental parameters and calculated from the analytes' calibration data (Fig. S1a–e, SI). Recovery values are within the range of 70–130%, which is the established acceptable range for the monitoring of environmental samples, including pharmaceuticals.^15,40^

**Fig. 5:**
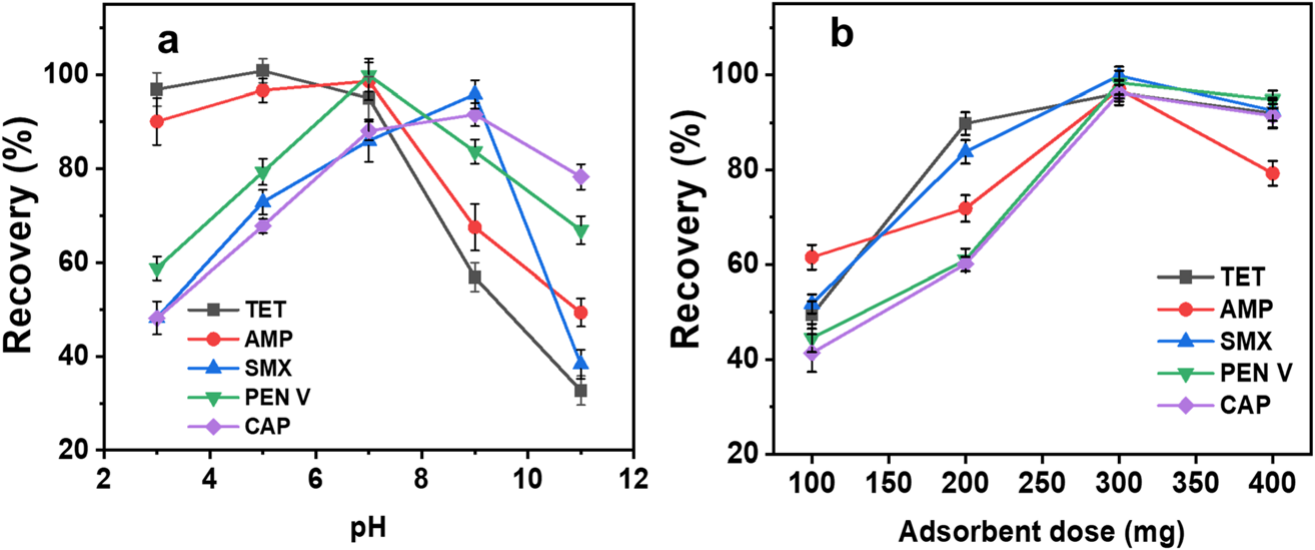
Effects of (a) pH and (b) adsorbent mass on the recovery of analytes.

The pH of the solution is an important parameter that can influence the surface charge of an adsorbent, dissociation of functional groups and the charge of some analytes.^26^Fig. 5a shows the effect of pH on the extraction recovery of TET, AMP, SMX, CAP and PEN V using the C/PVPP/MDI SPE sorbent. The result shows significant changes in the recovery as the solution pH moves from acidic to basic pH for all contaminants. For TET, AMP and PEN V, the recoveries decrease as the pH increases with a sharp decrease in recoveries at pH > 7.0. Fig. 5a shows that the maximum recovery for TET is at pH 5.0, whereas AMP and PEN V are at pH 7.0. From this result, we can infer the following:

1. Based on the speciation profile of TET, it exists primarily as neutral (H_2_TC^0^), anions (HTC^−^ and TC^2−^) or cations (H_3_TC^+^) within the pH range of 2.0–12.0.^41^ Within the pH range of 3.0–7.0, TET (p*K*_a_ 7.5–7.8) exists predominantly as neutral form because the dimethylammonium group begin to lose its proton and this could account for the high recoveries at this pH range. The extraction mechanism may involve hydrogen bonding interactions among the hydroxyl, amine, and carbonyl groups of TET, the hydroxyl groups present in cellulose, and the carbonyl groups of PVPP. Additionally, π–π interactions could occur between the aromatic rings of TET and MDI. However, in alkaline conditions, the anionic species of TET predominate due to the phenolic and tricarbonyl moieties' deprotonation (p*K*_a_ 9.2–9.7).^42^ The surface of C/PVPP/MDI is already negative at pH > 5.5 (pH_pzc_ = 5.5) and this results in electrostatic repulsion between the negatively charged TET molecules and C/PVPP/MDI. Therefore, this unfavourable interaction results in low recovery of TET at pH > 7.0.

2. For AMP, the primary amine, hydroxyl and carboxylic groups are the key functional groups. Based on its speciation profile, AMP (p*K*_a_*ca.* 7.2) exists primarily as a neutral molecule between pH 3.0–7.0.^43^ This accounts for the steady increase in recovery from pH 3.0 to 7.0. Similar to TET, the mechanism of extraction could be hydrogen bonding (between the carboxyl, amine and hydroxyl groups of AMP, hydroxyl groups of cellulose and carbonyl groups of PVPP), hydrophobic interactions (between the hydrophobic regions of AMP, PVPP and MDI) and/or π– π interactions (between the aromatic rings of AMP and MDI) Conversely, the anionic species of AMP predominates at higher pH values (pH > 7.0), which causes electrostatic repulsion between the negatively charged AMP molecules and the negatively charged C/PVPP/MDI molecules. As a result, recovery is drastically decreased at these pH values (Fig. 5a).

3. Similarly Fig. 5a thus shows that recovery of PEN V increases with increasing pH, with pH 7 showing the maximum recovery. PEN V primarily contains carboxylic acid and the amide nitrogen in the beta-lactam ring. Due to the protonation of the carboxylate group and the hydrolysis of the beta-lactam ring, PEN V is relatively unstable at pH values less than 4.0, and at pH values greater than 8.0, because of the deprotonation of the carboxylic group and the hydrolysis of the beta-lactam ring.^44,45^ The observed trend could be as a result of the hydrophobic interaction (between PEN V, isocyanate groups of MDI and hydrophobic regions of PVPP), hydrogen bonding (between hydroxyl groups of cellulose, carbonyl groups of PVPP and carboxyl, amine and amide groups of PEN V) as well as π– π interactions (between the aromatic rings of PEN V and MDI). In addition, the observed decrease in recovery above pH 7.0 may be attributed to repulsion between the anionic PEN V molecules and the negatively charged C/PVPP/MDI.

4. Fig. 5a illustrates that, for both CAP and SMX, recovery improves as the pH shifts from acidic toward basic conditions, reaching its peak at pH 9.0. This aligns with previous findings,^46^ which indicate that above pH 9.0, CAP predominantly exists as a negatively charged species rather than in a neutral molecular form, due to deprotonation of its phenolic hydroxyl group. From pH 5.5 onward, the extraction of CAP at pH 9.0 likely involves multiple mechanisms, including hydrophobic interactions among the hydroxyl groups and aromatic rings of CAP, the non-polar regions of PVPP, and the isocyanate groups of MDI. Additionally, hydrogen bonding may occur between CAP's chlorine, amide, and hydroxyl groups, the hydroxyl groups of cellulose, carbonyl groups of PVPP, and isocyanate groups of MDI. π–π interactions are also probable between the aromatic ring π electrons of CAP and MDI.

5. In contrast, SMX is relatively stable in mildly acidic conditions (pH < 5.0). Conversely, in neutral to slightly alkaline conditions (pH 7.0–9.0), SMX maintains a balance between its neutral form (caused by the aniline nitrogen) and anionic form (caused by the sulfonamide group starting to deprotonate). At high alkaline conditions (pH > 9.0), SMX primarily exists as an anionic species due to the deprotonation of the sulfonamide group.^47^ Possibly, the maximum recovery seen at pH 9.0 for SMX could be by a combination of mechanisms such as to hydrogen bonding (between the sulfonamide and amino groups of SMX, hydroxyl groups of cellulose and the carbonyl groups of PVPP), hydrophobic interactions (between the aromatic rings of SMX, non-polar regions of PVPP and isocyanate groups of MDI) and/or π– π interactions (between π electrons in the aromatic rings of SMX and MDI).

In addition to pH, the quantity of adsorbent plays a crucial role in practical applications. Given that this study targets the detection of analytes at trace levels, determining the minimum adsorbent mass required for effective extraction and maximum recovery is essential. Fig. 5b shows that for all five pollutants, the adsorbent dose is a significant variable in the extraction process. Higher extraction recovery is observed with increasing adsorbent mass up to 300 mg. This could be associated with the availability of more adsorption sites on the C/MDI/PVPP composite as the dose is increased. However, there is no further increase in recovery when the adsorbent mass increases from 300–400 mg; rather, a decline in recovery is observed at these higher doses. This could be due to the trapping of the pollutant molecules by the adsorbent which are not desorbed in the elution process. Hence, 300 mg is chosen as the optimum adsorbent dose for further experiments.

**Fig. 6:**
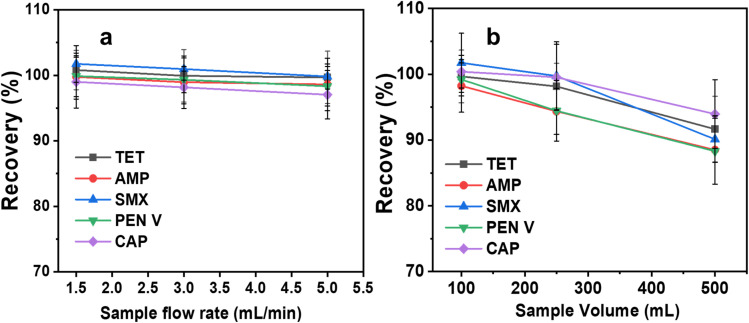
Effects of (a) sample flow rate and (b) sample volume on the recovery of analytes.

Apart from pH and adsorbent mass, selecting the optimal sample flow rate in SPE is critical, as it influences both the extraction recovery and the overall speed of the extraction procedure.^18^Fig. 6a shows that there is no increase in extraction recovery for the five pollutants within the investigated flow rates (1.5–5 mL min^−1^). However, for a rapid extraction process, 5 mL min^−1^ is selected as the optimum sample flow rate.

Fig. 6b shows the influence of sample volume on the extraction recovery. The enrichment factor (EF, which directly relates to extraction efficiency and % recovery) is enhanced by increasing the sample-to-eluent volume ratio. The results indicate that the percentage recovery remains largely unchanged between sample volumes of 100 and 250 mL, although a slight decline in recovery is observed when the volume increases to 500 mL. Even after increasing the sample volume to 500 mL, the lowest extraction recovery out of the five analytes (AMP, SMX and PEN V) is still *ca.* 90%. The EF of the C/PVPP/MDI calculated according to eqn (S6), SI is 63. In the case of evaporation and reconstitution of the eluate to 0.5 mL, the EF is 1000.

**Fig. 7:**
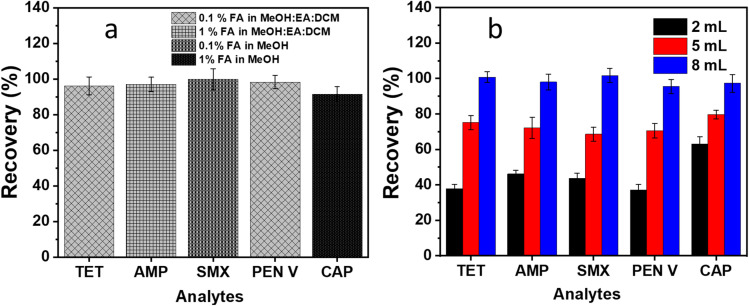
(a) Recovery of the analytes using the optimized elution solvents and (b) effect of the optimized elution solvents volume on the recovery of the analytes.

A crucial stage in the SPE process is effectively desorbing all or nearly all the analytes that have been adsorbed onto the adsorbent surface. Therefore, a good solvent for recovery provide a high desorption efficiency from the adsorbent and dissolve a high amount of the analyte in very little solvent volume.^26^ Single organic solvents such as methanol (MeOH), acetonitrile, ethyl acetate (EA), dichloromethane (DCM), isopropanol *etc.* could not achieve satisfactory elution of TET, AMP, SMX, PEN V and CAP from C/PVPP/MDI. In all cases, the recoveries were lower than 50% (Fig. S5, SI). Given the differing chemical properties of the antibiotics and their varying interaction mechanisms with the C/PVPP/MDI sorbent, a range of solvent mixtures and acidified solvents were investigated. As shown in Fig. 7a, the use of formic acid (FA) as a modifier significantly enhanced recovery. The optimum elution conditions for each analyte were determined as follows: 0.1% FA in MeOH for SMX, 1% FA in MeOH for CAP, 0.1% FA in MeOH : EA : DCM (1 : 1 : 1) for TET, and 1% FA in MeOH : EA : DCM (1 : 1 : 1) for PEN V and AMP. These results suggest that both solvent polarity and acidity influence desorption efficiency, likely by weakening hydrogen bonding and disrupting π–π or electrostatic interactions between the analytes and the adsorbent.

After determining the most suitable solvent for each analyte, the most effective volume of each elution solvent was also studied to obtain the highest recovery. Fig. 7b shows a similar trend for the recovery of the five analytes as an increase in solvent volume generally increases the recovery. The highest recovery is obtained by eluting with 8 mL of each optimum solvent.

**Fig. 8:**
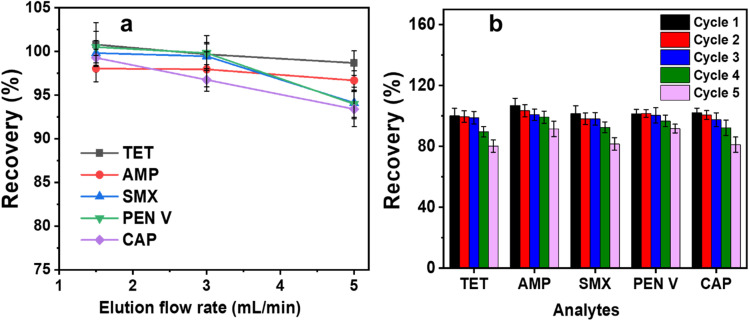
(a) Effect of elution flow rate and (b) reuse capacity of C/PVPP/MDI for the recovery of analytes in water.

Elution flow rate is a critical parameter in SPE, as it directly affects the contact time between the eluent and the sorbent material, which in turn influences analyte desorption and overall recovery. Generally, slower flow rates allow more interaction time, enhancing elution efficiency up to an optimal threshold; beyond this point, further decreases in flow rate may not yield significant improvements.^18^ As shown in Fig. 8a, increasing the elution flow rate from 1 to 5 mL min^−1^ had minimal effect on the recoveries of TET and AMP. However, there is a slight decrease in the recoveries of SMX, PEN V and CAP as the elution flow rate increases, suggesting that these analytes require slightly longer contact time with the eluent to achieve complete desorption. Given the relatively small elution volume used (8 mL), even at lower flow rates, the total elution time remains short. Therefore, an elution flow rate of 3 mL min^−1^ is selected as the optimal condition, balancing efficient analyte recovery with practical analysis time.

To evaluate the reusability of the adsorbent for extraction of the target analytes, five cycles of regeneration experiment were carried out (Fig. 8b). Fig. 8b shows that there is no significant difference in extraction efficiency after the first three reuse cycles for all the analytes. However, a slight reduction in recovery is observed after the fourth and fifth cycles. This decline may result from saturation of active sites with contaminant molecules, loss of adsorption sites during regeneration, structural fatigue or collapse of the C/PVPP/MDI sorbent, a common limitation in polymer-modified sorbents.^46^ While the use of regenerated SPE adsorbents is not recommended when high sensitivity and accuracy are critical, they can still be reused in monitoring applications as long as the recovery remains within 10% of the initial value.^15^ Therefore, the C/PVPP/MDI adsorbent is only suitable for five reuse cycles for AMP and PEN V, and four reuse cycles for TET, SMX and CAP after which the results may not be reliable.

### 3.3. Validation of the SPE method

Validating the limit of detection (LOD) and limit of quantification (LOQ) is a critical step in the SPE process, as it helps determine the suitability of the method for the expected concentration range of the target analytes. In this study, LOD and LOQ values were calculated based on the standard deviation (*n* = 6) of concentrations at selected low enrichment levels. Table 2 presents the LODs as 1.45, 0.05, 0.03, 2.07, and 1.84 ng L^−1^, and the LOQs as 4.40, 0.16, 0.09, 6.26, and 5.58 ng L^−1^ for TET, AMP, SMX, PEN V, and CAP, respectively. These low detection limits demonstrate the method's sensitivity and suitability for monitoring trace levels of these antibiotics in environmental water samples.

**Table 2:** Method validation for SPE of TET, AMP, SMX, PEN V and CAP by C/PVPP/MDI adsorbent

Analyte	Linear range (ng L^−1^)	*R* ^2^	LOD (ng L^−1^)	LOQ (ng L^−1^)	Spiked conc. (ng L^−1^)	Intra-day		Inter-day	
						Recovery (%)	RSD (%) *n* = 6	Recovery (%)	RSD (%) *n* = 6
TET	5.0–25.0	0.9990	1.45	4.40	5	96.3	9.6	103.5	4.7
					15	101.6	1.9	97.3	3.2
					25	100.4	3.8	98.6	2.7
AMP	0.25–5.0	0.9951	0.05	0.16	0.25	117.7	6.7	108.9	8.6
					1.0	91.2	0.4	97.3	2.2
					5.0	101.5	0.1	100.1	0.6
SMX	0.5–5.0	0.9932	0.03	0.09	0.5	119.6	1.9	103.4	7.8
					1.5	100.1	0.5	95.3	2.9
					5.0	102.2	0.9	94.6	2.1
PEN V	5.0–25.0	0.9941	2.07	6.26	5	101.6	9.7	94.9	10.1
					15	98.9	8.5	95.5	8.6
					25	100.3	2.9	98.6	5.9
CAP	5.0–25.0	0.9993	1.84	3.90	5	92.6	12.1	88.1	19.4
					15	97.6	7.2	86.5	18.6
					25	99.5	4.2	97.7	5.9

The linear range evaluates the performance of an analytical method by confirming that the calibration data remain linear across the expected concentration levels of the target analyte. For each analyte, a five-point calibration curve was established over a concentration range of 0.25 to 25 ng L^−1^ (Fig. S3a–e, SI). The calibration plots exhibit regression coefficients (*R*^2^) exceeding 0.99 in all cases (Table 2), demonstrating excellent linearity within the tested range. Considering the trace levels of these pollutants reported in water samples, the selected linear range covers the typical environmental concentrations of TET, AMP, SMX, PEN V, and CAP. Consequently, the observed linearity confirms the developed SPE method's suitability for analyzing these contaminants.

Precision describes the extent to which the results of repeated analysis of a given sample are consistent with each other. In this study, intra- and inter-day precision is reported using relative standard deviations (RSD) at three concentration levels. The intra- and inter-day precisions were lower than 20% for the five analytes (Table 2), which is considered acceptable for most complex samples and chromatographic methods.^48^

A recovery study is used to measure the accuracy of measurements in SPE method development, reflecting how close a measured value is to the true value. In this study, recoveries were measured at three concentration levels (Table 2) for each of the five analytes of interest. These three concentration levels were chosen to test the ruggedness of the developed method, as recovery may become unacceptable at very low concentrations. Table 2 shows that recoveries ranged from 91.2 to 119% for all five analytes, demonstrating that the SPE method is efficient. These recovery values fall within the acceptable range for pharmaceutical analysis in environmental water samples.^40^

### 3.4. Determination of pharmaceutical compounds in polluted water samples

To assess the applicability of the C/PVPP/MDI-SPE method for real environmental samples, recovery studies are conducted using groundwater and river water spiked with five target antibiotics (TET, AMP, SMX, PEN V, and CAP). The chromatograms of these samples are shown in the SI (Fig. S6). As presented in Table 3, the method achieved high recovery rates in both water types. In groundwater, mean recoveries are 94.3% (TET), 93.6% (AMP), 90.9% (SMX), 93.7% (PEN V), and 97.7% (CAP), while river water sample yields recoveries of 84.9%, 90.2%, 89.4%, 94.7%, and 92.6% for the same compounds, respectively. These values fall within the commonly accepted recovery range of 70–120% for trace-level SPE analysis in complex matrices.^40^ The evaluation in real samples is essential due to potential matrix interferences that can affect analyte recovery, and the results confirm the method's reliability for practical monitoring applications.

**Table 3:** Comparison of C/PVPP/MDI and commercial HLB SPE adsorbents for analyte recovery from spiked environmental water samples

Analytes	Spiked conc. (ng L^−1^)	Tap water		River water	
		Recovery (%)		Recovery (%)	
		C/PVPP/MDI	Commercial HLB	C/PVPP/MDI	Commercial HLB
TET	20.0	94.3	95.2	84.9	87.0
AMP	0.5	93.6	97.3	97.3	91.3
SMX	5.0	90.9	94.6	94.6	92.1
PEN V	20.0	93.7	94.7	95.8	93.3
CAP	5.0	97.7	92.6	92.6	89.8

Additionally, a direct comparison with a commercial hydrophilic–lipophilic balance (HLB) cartridge showed that the C/PVPP/MDI adsorbent delivered comparable recovery performance (Table 3). While commercial HLB adsorbents are well established for environmental analysis, their high cost is often prohibitive in developing countries, limiting widespread use. In contrast, cellulose, the most abundant natural polymer,^2^ offers a renewable, low-cost platform for synthesizing efficient SPE materials, as demonstrated in this study.

### 3.5. Performance comparison of C/PVPP/MDI with other SPE adsorbents

Considering the importance of monitoring antibiotics to ensure water safety and public health, various classes of SPE adsorbents have been developed to enable their extraction and quantification in environmental water samples. In this study, the performance of the C/PVPP/MDI adsorbent is compared with other SPE materials reported in the literature. As shown in Table 4, the recoveries and limits of detection (LODs) for TET, AMP, SMX, PEN V, and CAP using the developed method fall within the widely accepted range of 70–120% for trace environmental analysis.^40^ Many reported SPE adsorbents, while effective in some aspects, are limited by low recoveries, high detection limits, complex synthesis procedures, or limited reusability. In contrast, the C/PVPP/MDI adsorbent offers a balanced and practical alternative, exhibiting higher or comparable analytical performance across all five target antibiotics. For example, it achieved recovery rates above 90% for most analytes and sub ng L^−1^ detection limits in some cases, outperforming several reported materials in both sensitivity and efficiency (Table 4).

**Table 4:** Performance comparison of C/PVPP/MDI with other SPE adsorbents

Adsorbent	Contaminant	Type of water	Linear range (ng L^−1^)	Analysis method	Recovery (%)	LOD (ng L^−1^)	Reference
Magnetic activated carbon from rice husk	TET	Distilled water	500–100000	LC-MS/MS	95.0–96.0	100–950	49
Graphene oxide	TET	Tap, river and wastewater	20–100 000	LC-MS/MS	84.2–105.5	2.4–25.2	50
Graphene oxide	AMP	Tap water	500–200000	HPLC-UV	96.4–101.6	40	51
Magnetic hypercrosslinked polystyrene	SMX	River water	2000–200000	HPLC-UV	84.0–105.0	210–330	52
Fe_3_O_4_@SiO_2_@MIPs	SMX	Tap, river, lake and hospital wastewater	10–1000	LC-MS	73.8–96.2	1.4–2.8	53
CMDI	CAP	Tap and river water	500–8000	HPLC-UV	86.8–96.2	71.0	15
Molecularly imprinted microspheres	CAP	Seawater	10–100	HPLC-DAD	81.0–90.0	5.0	54
C/PVPP/MDI	TET	Tap and river water	5–25	LC-MS/MS	84.9–94.3	1.45	This study
	AMP		0.25–5		90.2–93.6	0.05	
	SMX		0.5–5		89.4–90.9	0.05	
	PEN V		5–25		93.7–94.7	2.07	
	CAP		5–25		92.6–97.7	1.84	

The present method offers several advantages: (1) a facile synthesis of the adsorbent using a low-cost and naturally abundant polymer precursor (cellulose), (2) high analyte recoveries, (3) low LODs suitable for environmental monitoring, and finally, (4) a simple and rapid extraction procedure. These features position the developed SPE method as a viable alternative to more complex or costly adsorbents, especially in resource-limited settings.

## 4. Cost economics of adsorbents

A key objective of this study is to develop a cost-effective SPE adsorbent capable of efficiently extracting contaminants such as TET, AMP, SMX, PEN V and CAP from water. The cost of the adsorbent is key in determining its commercial applicability and scalability for real life situations. Because of the importance of cost, we estimate that the total cost for the synthesis of 1 kg of C/PVPP/MDI SPE adsorbent is approximately €708 (Table 5). Therefore, the estimated cost of 300 mg of C/PVPP/MDI SPE adsorbent is approximately €0.21. A pack of 50 cartridge containers costs around €54, while producing a pack of 50C/PVPP/MDI SPE adsorbents costs about €65. Including labor costs (approximately €50), the total production cost for a 50-pack of SPE adsorbents is roughly €115. Even if we estimate a margin for the producing company of €50, the total estimated cost of €165 for a 50-pack of C/PVPP/MDI SPE adsorbents remains significantly lower than that of commercially available HLB SPE sorbents, which typically exceed €300 for similar quantities (excluding shipping). For example, a pack of 30 SPE tubes containing 300 mg of C/PVPP/MDI adsorbent, costing about €99, is nearly 50% less expensive than a comparable pack of 200 mg Oasis HLB sorbents, priced at €198 (https://www.analytics-shop.com/de/wtwat106202, accessed 21st July 2025).

**Table 5:** Cost estimation for the preparation of 1 kg of C/PVPP/MDI adsorbent we definitely need references for these values

Activity	Sub-sections	Break-down	Cost (€)	Price (€/Amount)
Material processing	Cellulose	1 kg of cellulose	134	3.3/25 g
Cellulose cross-linking	MDI	1 kg of MDI	145	3.6/25 g
	PVPP	1 kg of PVPP	202	5.1/25 g
	Solvothermal treatment and drying cost	Power of oven (0.76 kWh) * run time (h) * unit cost per kWh (0.29 euros) 1 L of DMF	2.6	
	Solvent cost		60	5.9/100 mL
Labor cost			100	
Net cost			643.6	
Overhead cost (@ 10% of net)			64.36	
Total cost			707.98	

## 5. Limitations of the current method

While the developed C/PVPP/MDI SPE adsorbent shows excellent performance for selected antibiotics, some limitations remain. First, the material's selectivity for other classes of emerging contaminants, including non-antibiotic pharmaceuticals and micro-pollutants, has not been fully assessed. This aligns with a recent review highlighting that even advanced cellulose-based SPE materials are primarily evaluated for specific groups of pollutants rather than a broader spectrum.^2^ Second, while the synthesis process is relatively simple and low-cost at laboratory scale, its scalability and consistency in larger production batches remain to be verified. Indeed, studies on cellulose adsorbents have repeatedly emphasized that scalability and technical challenges need to be considered for large-scale production.^55,56^ Third, although a preliminary time-dependent adsorption screening was conducted, a detailed investigation of mass transfer kinetics, important for understanding adsorption mechanisms and potential rate-limiting steps, was beyond the scope of this study. Future research should therefore focus on these aspects to strengthen the method's applicability for large-scale, cost-effective monitoring, especially in resource-limited settings.

## 6. Conclusion

This study introduces a low-cost, cellulose-based SPE adsorbent (C/PVPP/MDI) for the effective preconcentration and determination of multiple antibiotics: TET, AMP, SMX, PEN V, and CAP in environmental water. The key innovation lies in the strategic integration of cellulose, PVPP, and MDI to create a chemically robust, stable and reusable sorbent capable of extracting both hydrophilic and hydrophobic pharmaceuticals with high efficiency. The developed SPE/LC-MS method demonstrated excellent analytical performance, with low detection limits (0.03–2.07 ng L^−1^), strong linearity (*R*^2^ > 0.99), and high recovery rates (84.8–97.6%), closely matching commercial HLB cartridges. Importantly, the adsorbent retained its performance over at least five reuse cycles and costs approximately 50% less than commercial alternatives, offering a scalable and practical solution for resource-limited settings. Overall, this work highlights the potential of modifying renewable biopolymers like cellulose for use in affordable and high-performing analytical tools. In addition, the C/PVPP/MDI adsorbent provides a promising platform for environmental antibiotic monitoring and contributes a meaningful advancement toward accessible water quality assessment in developing regions.

## Conflicts of interest

There are no conflicts to declare.

## Supplementary Material

RA-015-D5RA02296G-s001

## Data Availability

All data are available upon reasonable request to the corresponding authors. Supplementary information: The SI contains all data in a graphic form. See DOI: https://doi.org/10.1039/d5ra02296g.
