# Supplementary material for: A low-cost cellulose-based composite as an efficient solid phase extraction sorbent for the determination of antibiotics in water
Source: RSC Adv. 2025 Sep 10;15(39):32796–809. doi: 10.1039/d5ra02296g (PMC12422246; doi:10.1039/d5ra02296g)
Supplement: RA-015-D5RA02296G-s001 [file RA-015-D5RA02296G-s001.pdf]

Supplementary Information (SI) for RSC Advances.
This journal is © The Royal Society of Chemistry 2025

Electronic Supplementary Information

Low-cost cellulose-based composite as an efficient solid phase extraction sorbent for the determination of antibiotics in water

Damilare Olorunnisola[1,2,3,4], Chidinma G. Olorunnisola[1,3], Christina Günter[5], Chukwunonso P. Okoli[3,6], Martins O. Omorogie[3,4], Emmanuel I. Unuabonah[3,4], Harshadrai M. Rawel[2*], Andreas Taubert[1*]

[1] *Institute of Chemistry, University of Potsdam, D-14476 Potsdam, Germany*

[2] *Institute of Nutritional Science, University of Potsdam, 14558 Nuthetal, Potsdam, Germany*

[3] *African Centre of Excellence for Water and Environment Research (ACEWATER), Redeemer's University, PMB 230, Ede, Osun State, Nigeria.*

[4] *Department of Chemical Sciences, Redeemer's University, PMB 230, Ede, Osun State, Nigeria.*

[5] *Institute of Geosciences, University of Potsdam, D-14476 Potsdam, Germany.*

[6] *Department of Chemistry, Alex Ekwueme Federal University Ndufu-Alike, Ebonyi State, Nigeria*

Corresponding Author

Andreas Taubert ataubert@uni-potsdam.de

Harshadrai M. Rawel rawel@uni-potsdam.de

Table S1. LC-MS/MS operation conditions

MS/MS	
Ionization source	ESI positive or negative
Detection	Multiple reaction monitoring (MRM)
Fragment voltage	70 – 100 V; individually optimized
Collision energy (ev)	5 – 43 eV; individually optimized
Dwell time	50 ms
Sheath gas and temperature	Nitrogen, at 275 ^{o}C

Table S2. MS/MS condition for the identification of targeted compounds

Compound	Retention time (min)	ESI mode	Precursor ion (m/z)	Product ions (m/z)	Fragment voltage (V)	Collision energy (eV)
Tetracycline	4.9	+	445.2	427.1/410.1/153.9	80	10/20/28
Sulfamethoxazole	6.0	+	254.3	156/108	80	15/27
Ampicillin	5.5	+	350.1	192/174/160	80	16/16/25
Penicillin V	7.9	+	351.4	192/160/114	80	5/15/37
Chloramphenicol	7.1	+	324.1	306/275.9/165.9	70	6/15/35

S1.0 Analytes' Calibration Curve

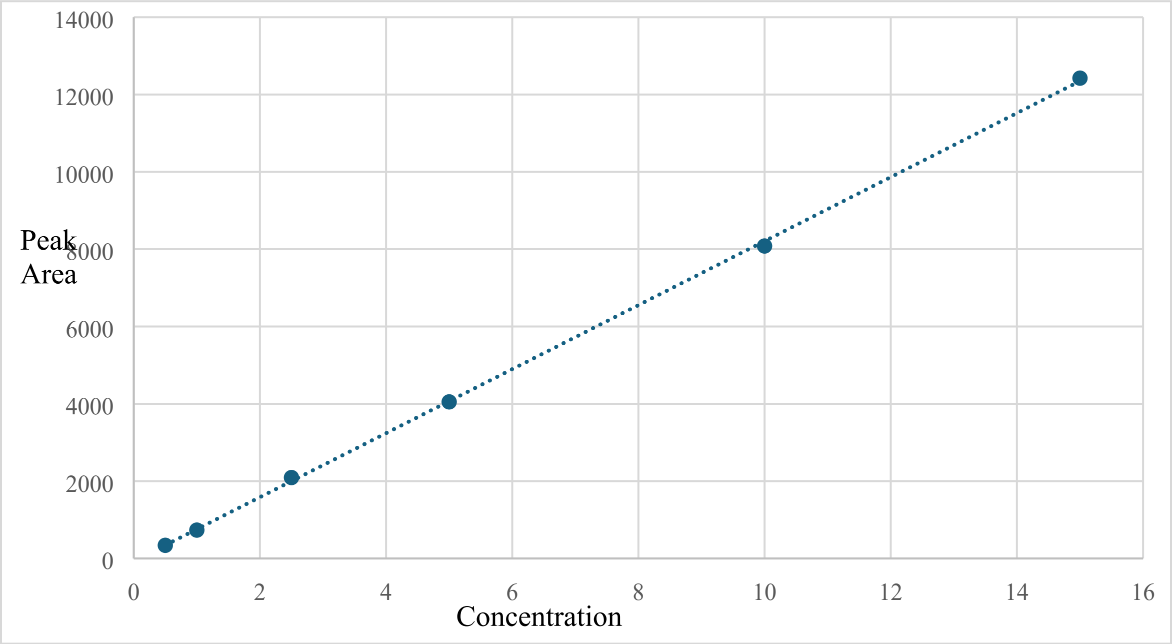

Fig S1a. Calibration curve to estimate Tetracycline (TET) recovery

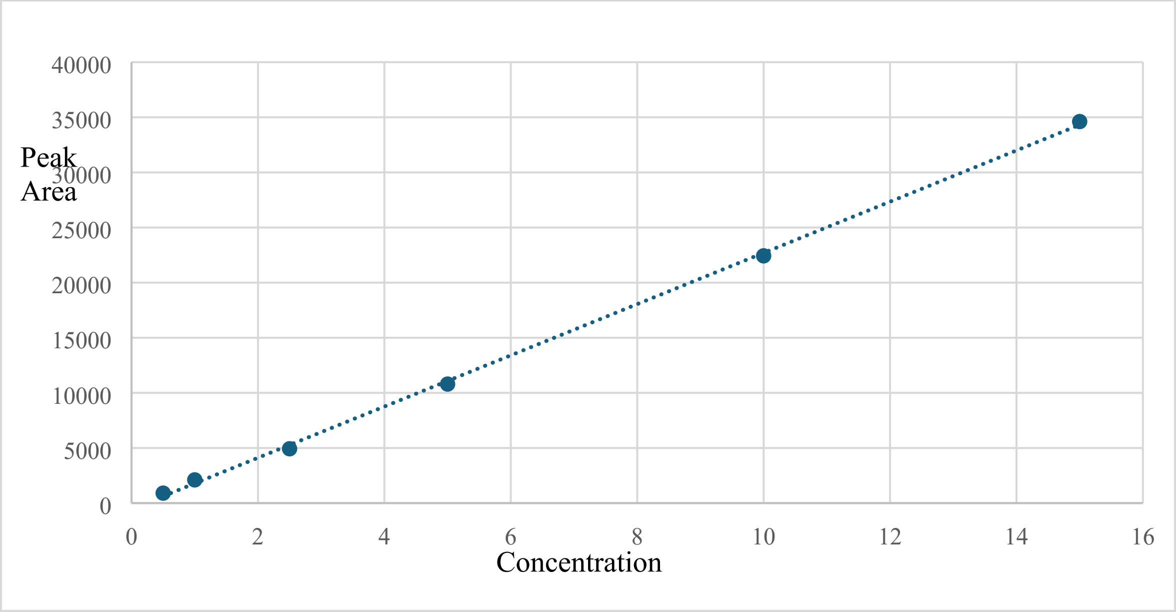

Fig S1b. Calibration curve to estimate Ampicillin (AMP) recovery

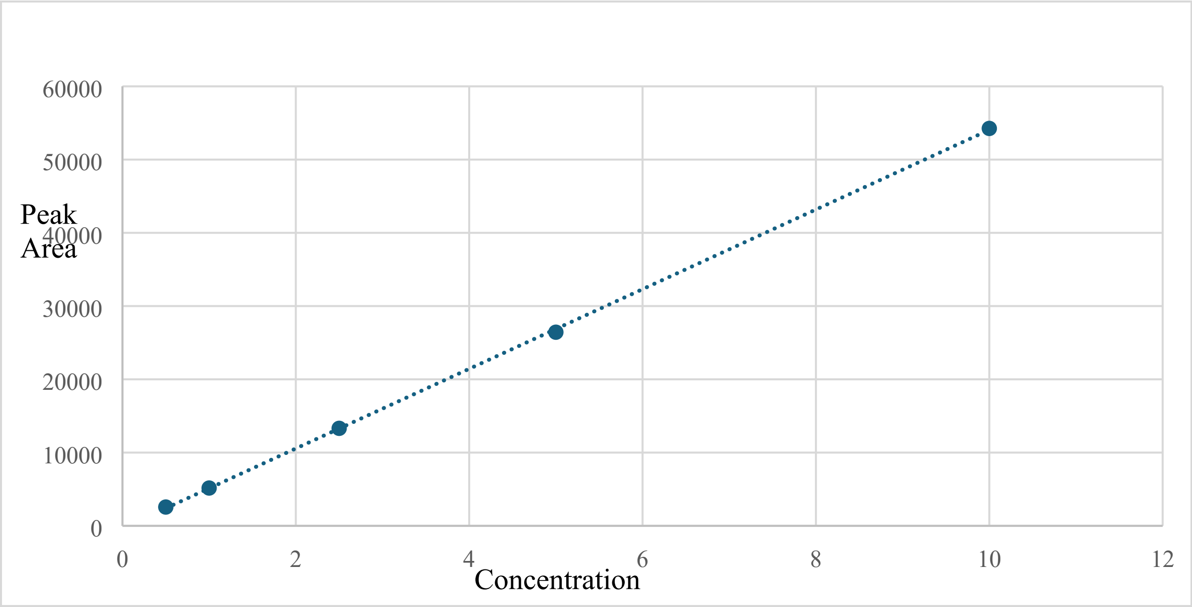

Fig S1c. Calibration curve to estimate Sulfamethoxazole (SMX) recovery

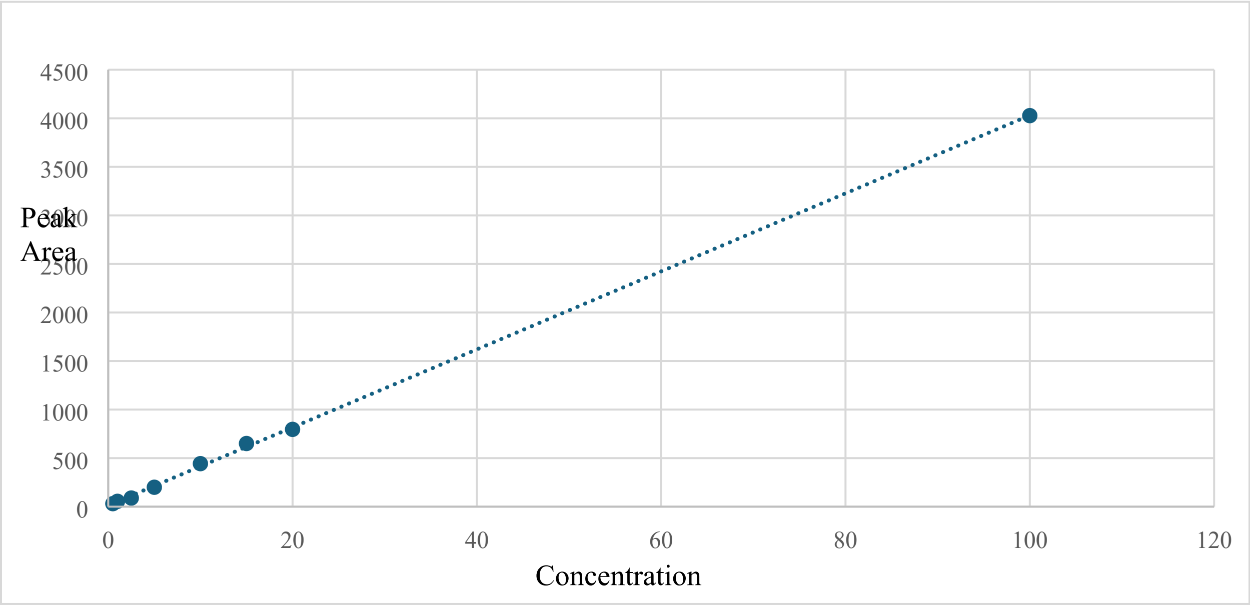

Fig S1d. Calibration curve to estimate Penicillin V (PEN V) recovery

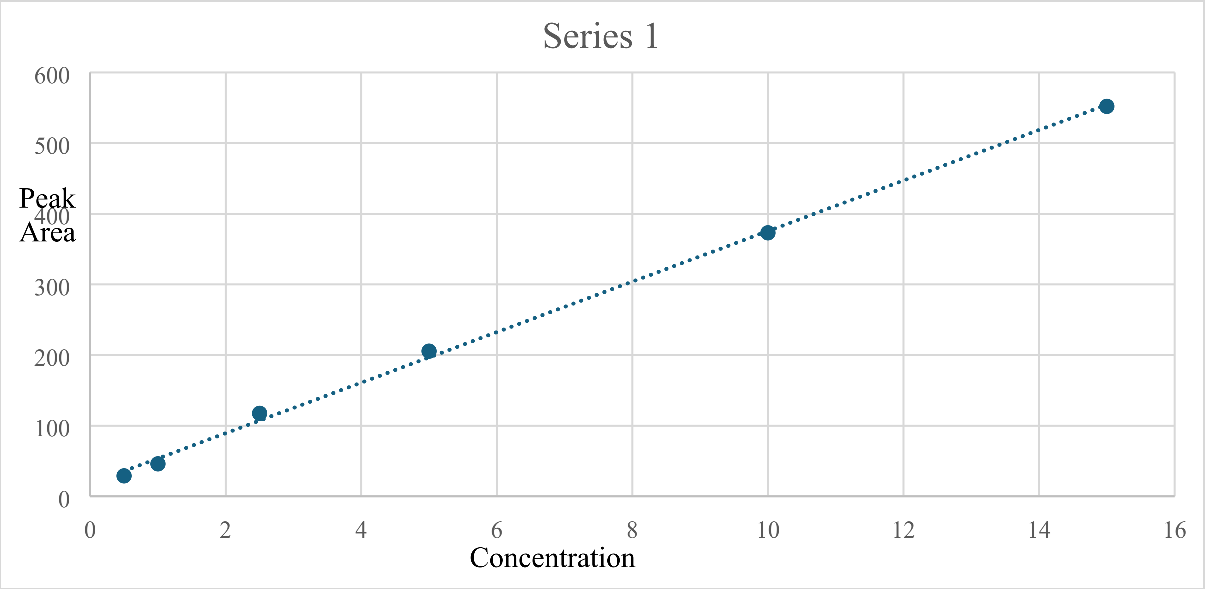

Fig S1e. Calibration curve to estimate Chloramphenicol (CAP) recovery

S2.0 Evaluation of Method Performance and Validation

The performance of the developed pre-concentration method was assessed by evaluating linearity/linear range, recovery, precision, and detection limit. These parameters were evaluated according to guidelines for single-laboratory validation of analytical methods for trace-level concentrations of organic chemicals, (Dayananda et al., 2015; Fajgelj & Ambrus, 2000) while validation was based on parameters defined in standard protocols describing chromatographic methods (Kaza, Karaźniewicz-Łada, Kosicka, Siemiątkowska, & Rudzki, 2019). The extraction efficiency (%) for each analyte was determined as:

$$\% R = \frac{C_e \times V_e}{C_s \times V_s} \times 100 \quad \text{(S1)}$$

Here, C_e is the concentration of eluate (ng/L), V_e the volume of eluate (mL), C_s the concentration of sample (ng/L) and V_s the volume of sample (mL).

The performance evaluation and validation of the parameters were investigated by enriching real and spiked water samples at suitable spike levels under the optimized SPE conditions.

Limit of detection (LOD) was evaluated by the official method according to the international conference on harmonization of technical requirements for registration of pharmaceuticals for human use (Guideline, 2013). LOD was calculated based on the standard deviation of the response and the slope expressed as:

$$LOD = \frac{3.3\,\sigma}{S} \qquad \text{(S2)}$$

where σ is the standard deviation of the response and *S* is the slope of the linear range calibration curve. Six replicate analysis of tap water and river water samples were done with different spike level. Limit of Quantification (LOQ) is expressed as:

$$LOQ = \frac{10\,\sigma}{S} \qquad \text{(S3)}$$

Precision was measured with relative standard deviations (RSD) of the data obtained from the experiment for MDL. RSD was calculated using the following expression:

$$RSD = \frac{s}{\bar{x}} \times 100 \qquad \text{(S4)}$$

where s is the standard deviation, and $\bar{x}$ is the mean value of six replicate measurements.

Linearity/linear range was evaluated by subjecting water samples containing the analytes in the concentration range 0.25 – 25 ng/L to extraction, pre-concentration and analysis.

The recovery study was done by using laboratory tap water and polluted water samples collected from the Ede river. Recovery was done by analyzing the un-spiked and spiked water samples, and the percentage recovery was calculated as follows:

$$Recovery = \frac{C_{sp} - C_{up}}{SL} \times 100\% \qquad (S5)$$

where C_{sp} is the concentration of the spiked sample (ng/L), C_{up} is the concentration of the unspiked sample (ng/L), and *SL* is the spike level.

Enrichment factor (EF) is defined as the ratio of volume of sample to volume of eluent:

$$EF = \frac{Vs}{Ve} \qquad (S6)$$

where *Vs* is the volume of sample and *Ve* is the volume of eluent.

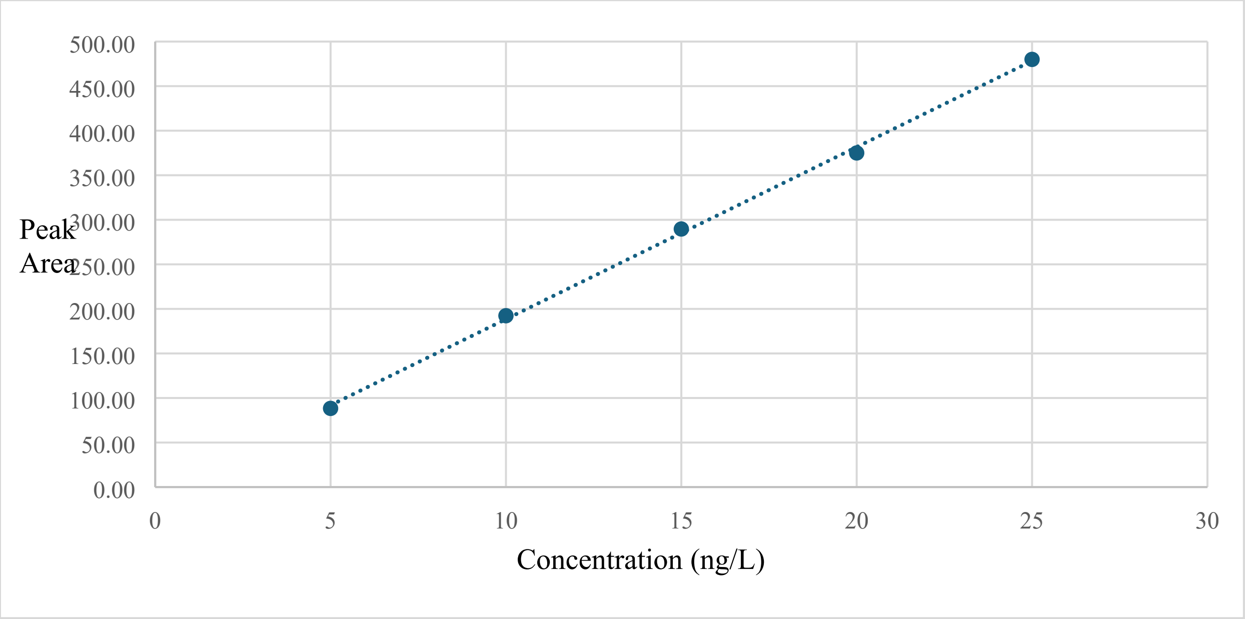

Fig S2a. TET linear range data for method validation

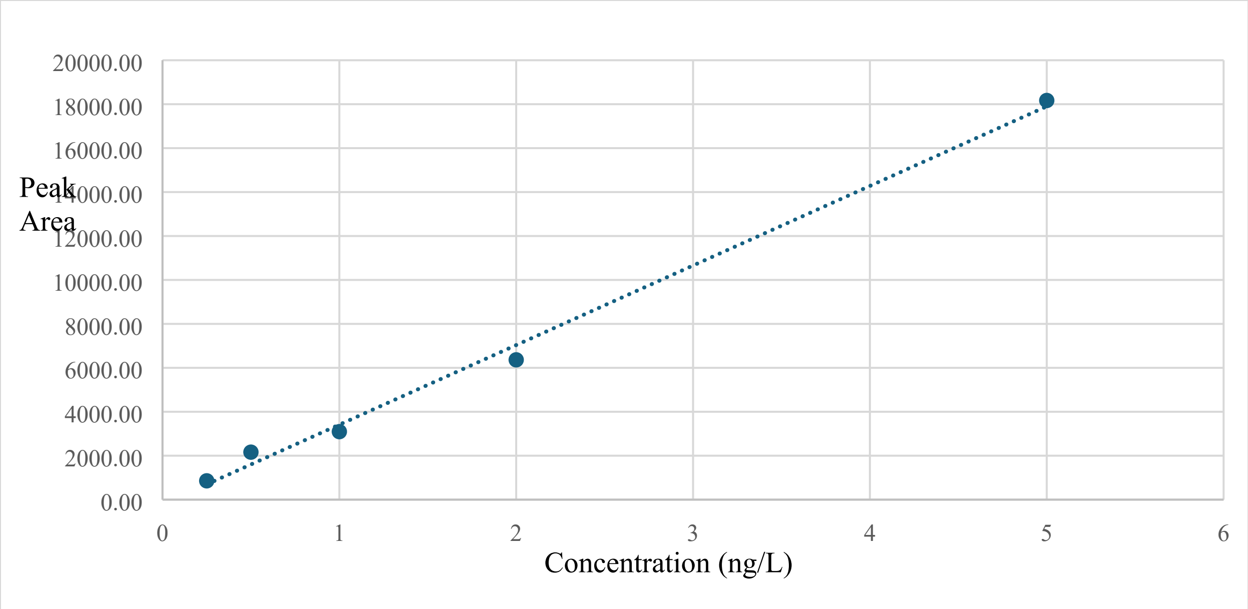

Fig S2b. AMP linear range data for method validation

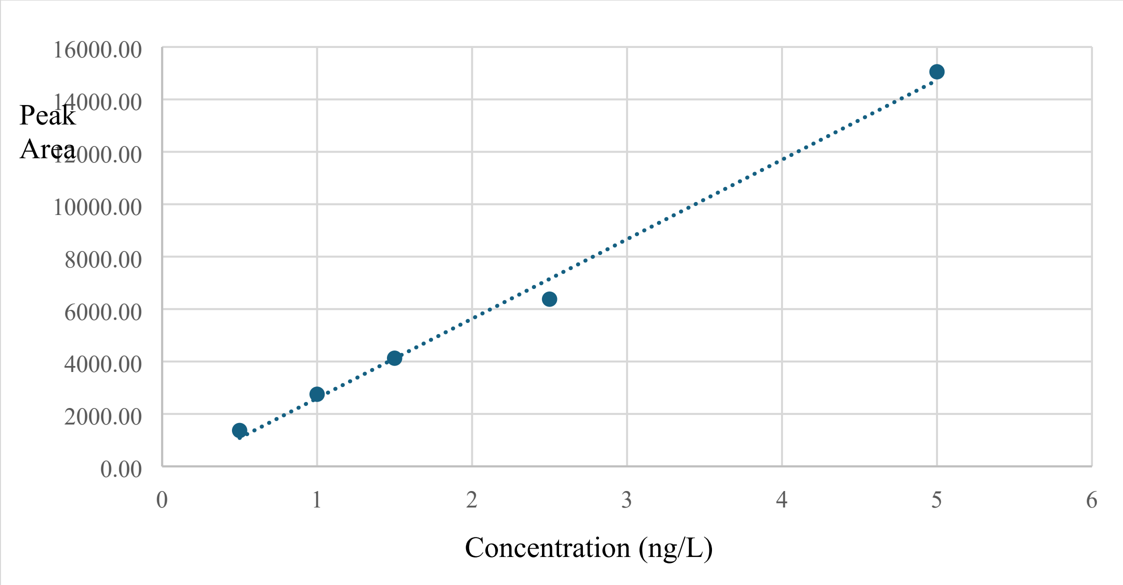

Fig S2c. SMX linear range data for method validation

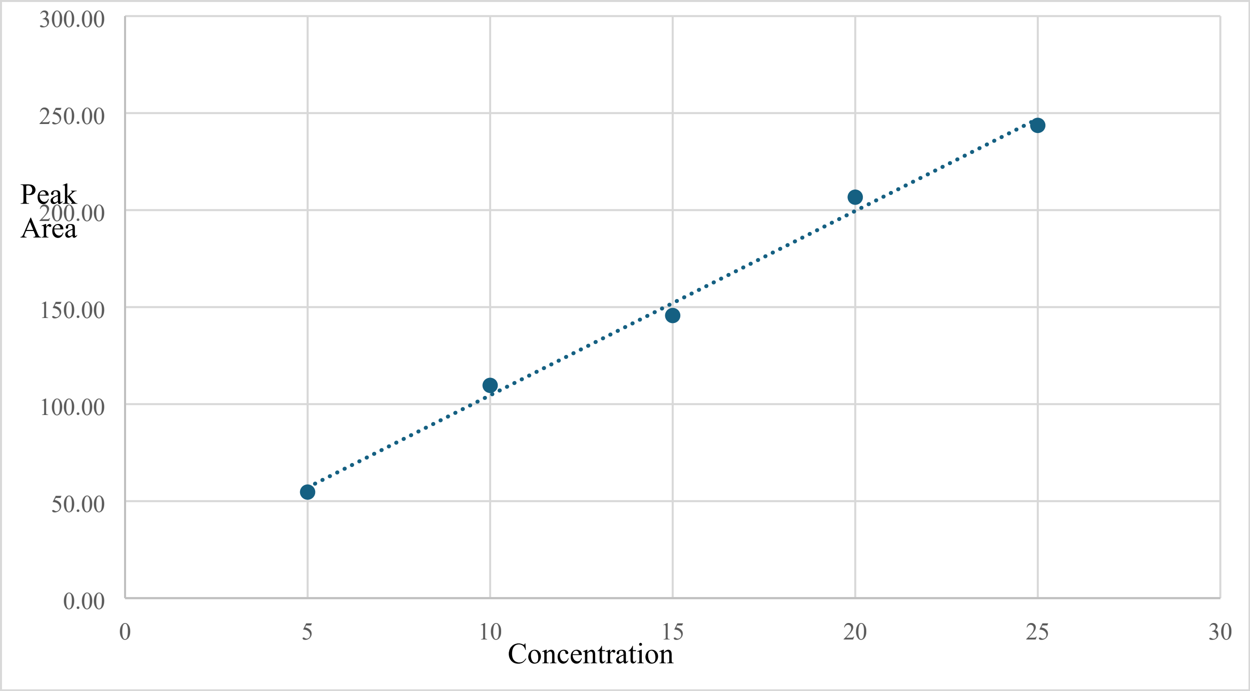

Fig S2d. PEN V linear range data for method validation

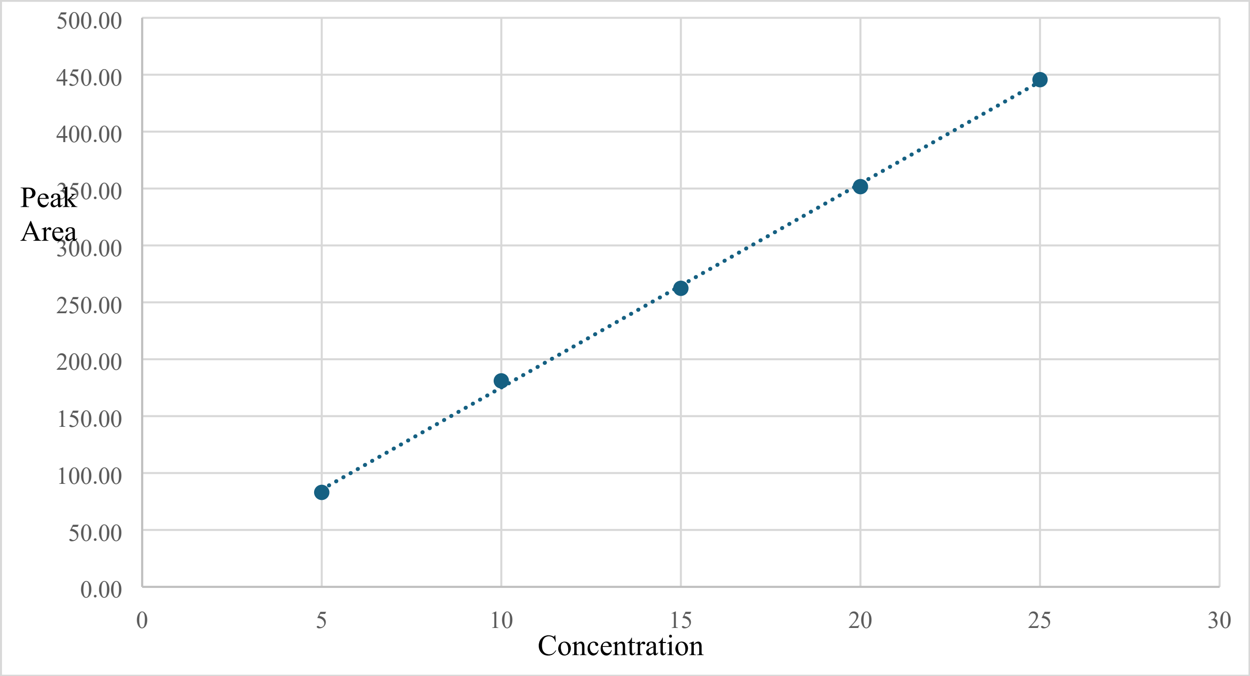

Fig S2e. PEN V linear range data for method validation

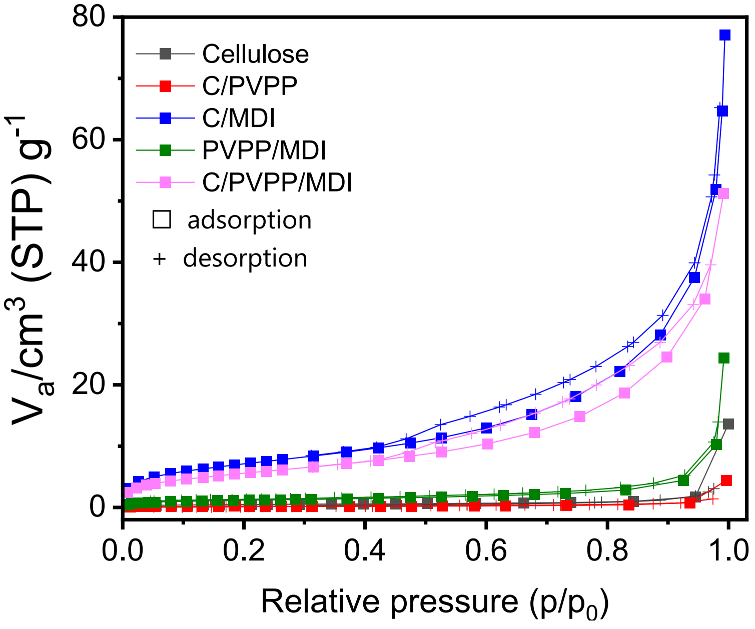

Fig. S3. BET plot for the prepared adsorbents

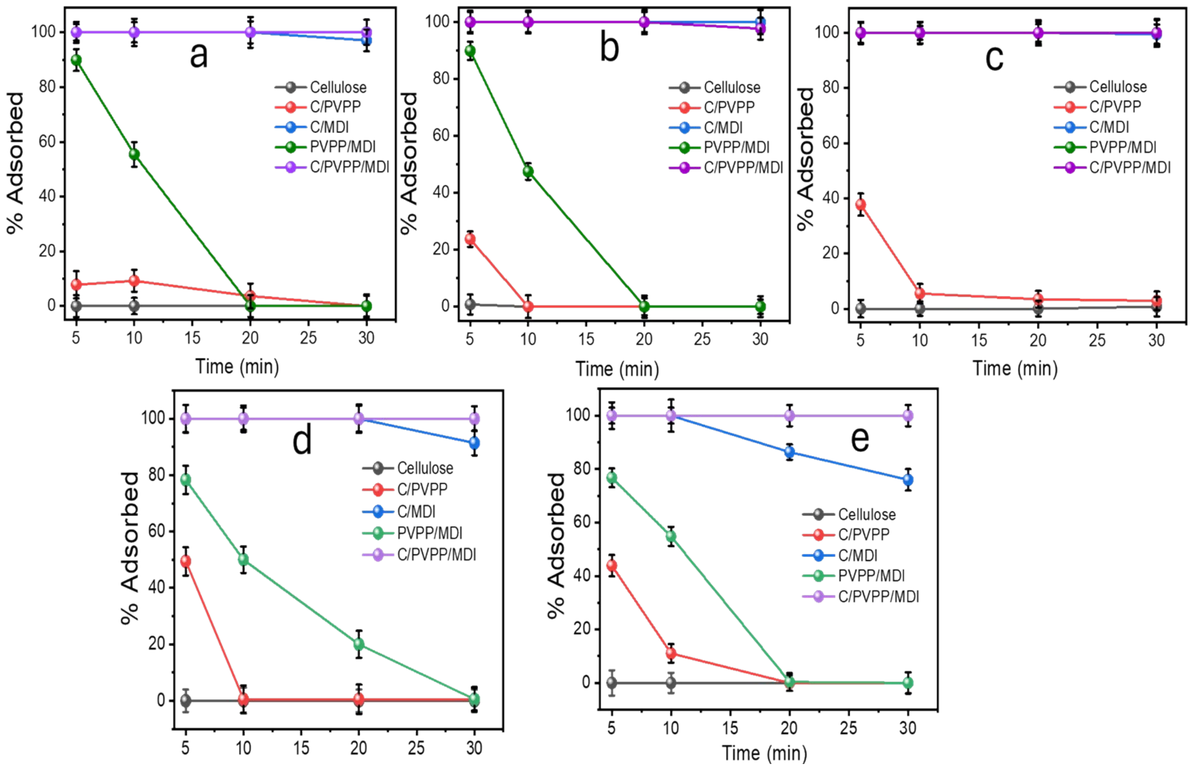

Fig S4. Result from the preliminary adsorption study of (a) TET (b) AMP (c) SMX (d) CAP and (e) PEN V (adsorbent dose:300 mg, contaminant concentration:5 ng/mL, sample volume: 150 mL).

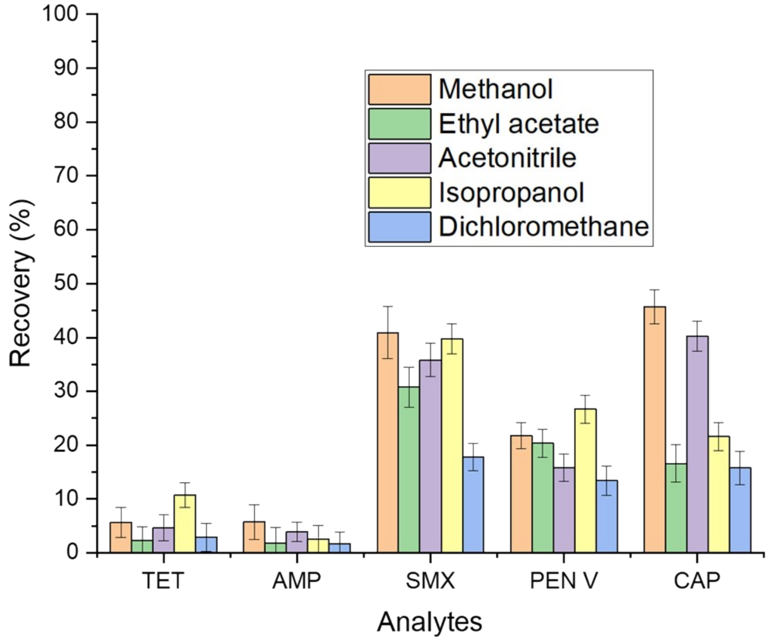

Fig S5. Effects of elution solvents on analytes' recovery

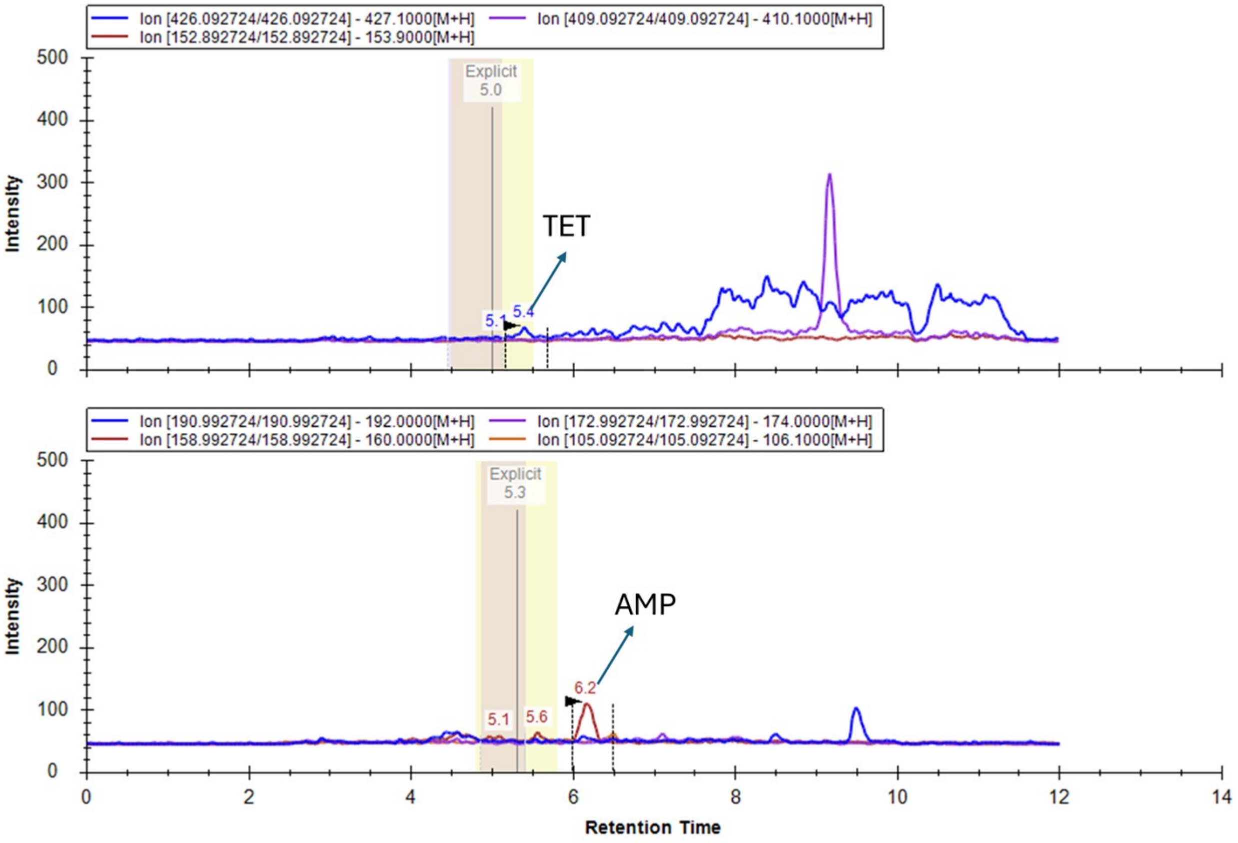

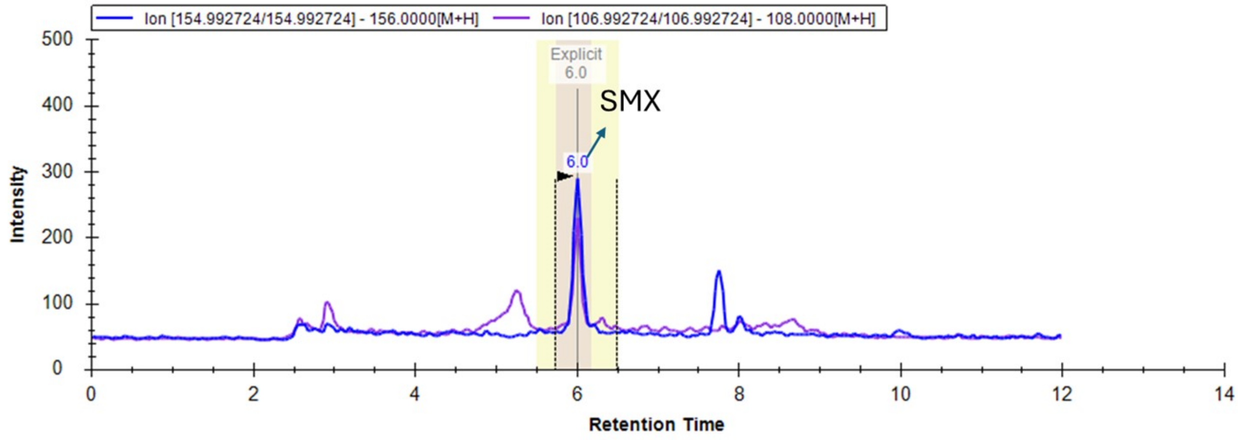
Ion [154.992724/154.992724] - 156.0000[M+H]
Ion [106.992724/106.992724] - 108.0000[M+H]
Explicit
6.0
SMX
6.0
Intensity
Retention Time
0
100
200
300
400
500
0
2
4
6
8
10
12
14

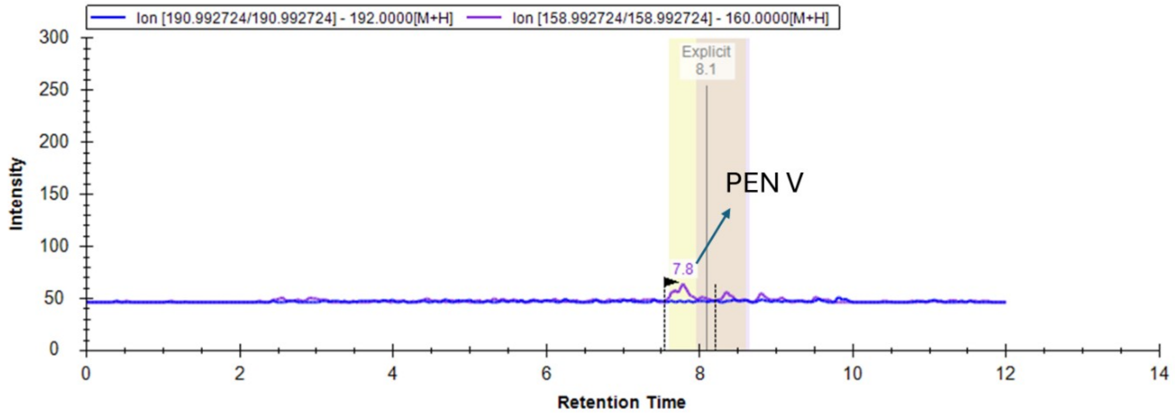
Ion [190.992724/190.992724] - 192.0000[M+H]
Ion [158.992724/158.992724] - 160.0000[M+H]
Explicit
8.1
PEN V
7.8
Intensity
Retention Time
0
50
100
150
200
250
300
0
2
4
6
8
10
12
14

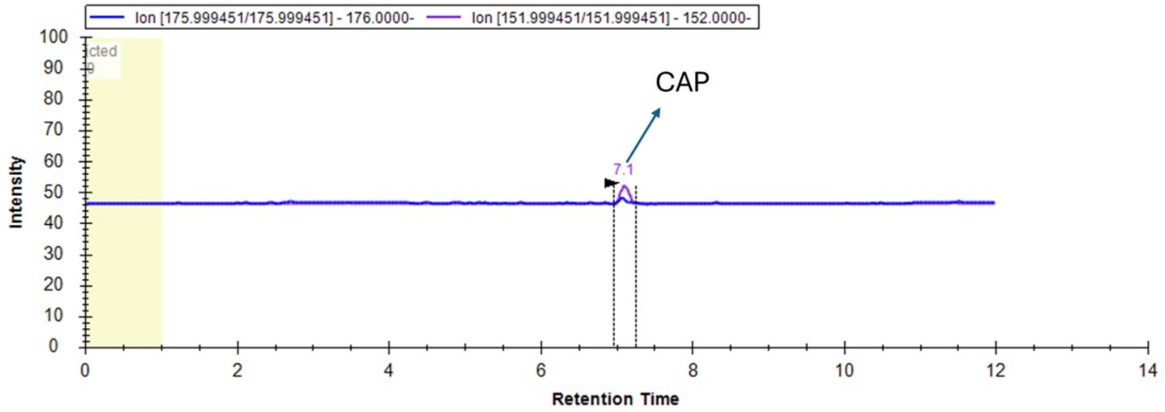

Fig S6. The chromatograms for the recovery of the analytes in real water sample
